# Single-cell consequences of X-linked meiotic drive in stalk-eyed flies

**DOI:** 10.1371/journal.pgen.1011816

**Published:** 2025-09-18

**Authors:** Peter D. Price, Sylvie M. Parkus, Victoria J. Lloyd, Ben T. Alston, Sasha L. Bradshaw, Sadé Bates, Margaret A. Hughes, Steve Paterson, Terry Burke, Iulia Darolti, Andrew Pomiankowski, Alison E. Wright

**Affiliations:** 1 Ecology and Evolutionary Biology, School of Biosciences, University of Sheffield, Sheffield, United Kingdom; 2 Department of Genetics, Evolution and Environment, University College London, London, United Kingdom; 3 Department of Integrative Biology, University of California, Berkeley, California, United States of America; 4 Centre for Genomic Research, University of Liverpool, Liverpool, United Kingdom; 5 Department of Ecology and Evolution, University of Lausanne, Lausanne, Switzerland; Peking University, CHINA

## Abstract

Sex-linked meiotic drivers limit the inheritance of the alternate sex chromosome in the heterogametic sex, subsequently skewing the offspring sex ratio. They consequently have large impacts on genome evolution, adaptation, and the emergence and maintenance of sexually selected traits. Despite this, our understanding of their molecular basis and consequences for gametogenesis and sex chromosome regulation more broadly has focused on a handful of model organisms, primarily *Drosophila* and mouse, which are not representative of the broad diversity of reproductive modes and drive systems in nature. Here, we employ single-cell RNA sequencing (scRNA-seq) to investigate a sex-linked meiotic driver in the Malaysian stalk-eyed fly, *Teleopsis dalmanni*. First, we produce a comprehensive single-cell atlas of the male *T. dalmanni* gonad and identify major testis cell types. We then provide a comprehensive profile of the cellular and transcriptional landscape of the testis, providing evidence for a lack of complete meiotic sex chromosome inactivation and complex trajectory of dosage compensation. Second, by contrasting single-cell expression data between drive and standard testes, we provide insight into the consequences of a meiotic driver for the transcriptomic landscape of the testis and sex chromosome regulation. Importantly, we show that the presence of a meiotic driver does not perturb fundamental patterns of X-linked regulation. Our results provide insight into how the meiotic driver might bias its transmission to the next generation and highlight genes with perturbed expression as a potential consequence of the disruption of spermatogenesis.

## Author summary

Meiotic drivers, a class of selfish gene, are frequently located on sex chromosomes and have dramatic impacts on gamete development. However, our understanding of their molecular consequences for gamete production and sex chromosome regulation has focused on a handful of model organisms. In this study, we use single-cell RNA-sequencing approaches to produce a single-cell atlas of the testis of the stalk-eyed fly, *Teleopsis dalmanni*. This species harbours an X-linked meiotic driver where drive males produce more than 90% female offspring. First, we generate a comprehensive profile of the cellular and transcriptional landscape of spermatogenesis. We show limited evidence for meiotic sex chromosome inactivation and unique patterns of dosage compensation across spermatogenesis, relative to both other dipterans and insects in general. Finally, by comparing single-cell expression data between standard and drive males, we show that although there are significant differences in genome regulation, broad expression dynamics in the testis are conserved in the presence of meiotic drive. Notably, we highlight key genes with perturbed expression as a potential consequence of the disruption of spermatogenesis by the X-linked meiotic driver.

## Introduction

Following Mendelian genetics, the expectation at meiosis is that maternal and paternal alleles segregate equally. However, meiosis is often a battleground for inheritance. Intragenomic conflicts emerge through selfish genetic elements forcing unequal segregation of alleles, skewing their chances of being represented in the mature germline [1–3]. These selfish genes, known as meiotic drivers, are widespread across eukaryotic life [4–8], and have large consequences for the ecology and evolution of populations [2,9–11].

Sex chromosome meiotic drivers are the most detectable form of meiotic drive [12] as they skew offspring sex ratios from 1:1. X-linked drivers reduce the inheritance of the Y chromosome and so have profound effects on reproductive traits [13,14], genome evolution [15–18], adaptation [19,20], sexual selection [21–23], and population persistence [1,9,20]. As a result, there has been considerable interest in manipulating drive systems for pest and disease eradication schemes [24]. Characterising the molecular mechanisms of meiotic drivers and their consequences is therefore key to understanding a range of biological processes.

However, despite meiotic drive having been identified almost a century ago [25], the evolutionary origins and the mechanisms by which drivers operate remain unclear. Furthermore, at the molecular level, they have been well studied in only a small handful of species, including *Drosophila* [26], *Anopheles* [27,28], house mouse [29–31], monkeyflower [5], yeast [32,33] and *Neurospora* [34]. This work highlights that diverse mechanisms are utilised by drivers to disrupt meiosis, including those that affect segregation at meiosis or motility of sperm, and that these can operate at distinct time points, including meiotic-acting and post-meiotic drivers [26,35,36]. However, given these findings are limited to only a handful of organisms, the generalities or lack thereof of these patterns is unclear.

One of the reasons that meiotic drivers have been studied in so few species is their complex genomic architecture. Meiotic drive involves interactions between at least two loci, a *driver* locus with driving and non-driving alleles and a *target* locus with alleles that are either resistant or sensitive to the *driver* [2]. To prevent the formation of suicidal haplotypes bearing both the driving allele and sensitive target allele [19] meiotic drivers are frequently housed by inversions [2]. These reduce recombination with the wildtype chromosome and guard against the subsequent breakup of their complex molecular phenotypes [6,17,37,38]. The resulting high level of linkage disequilibrium between the driver and neutral variation across these inversions limits the use of traditional genetic mapping approaches to identify the driver locus [19]. In addition, the processes that meiotic drivers disrupt, such as spermatogenesis, are complex and operate alongside unique regulatory mechanisms acting on the sex chromosomes, including dosage compensation and meiotic sex chromosome inactivation (MSCI), that are often poorly characterised in non-model organisms. In particular, X chromosome expression dynamics in the gonad are somewhat in dispute across many organisms, with a lack of consensus on the status of both regulatory processes [39–43].

Transcriptomics provides an important avenue for understanding both the unique regulatory processes operating in the testis during spermatogenesis and the molecular underpinnings and consequences of meiotic drivers. Notably, recent advances in single-cell expression approaches afford us a high-dimensional perspective of the impacts of meiotic drivers on the gonad, gametogenesis, and sex-linked expression. This is because they provide fine-scale data on regulatory variation at single-cell resolution as well as data on the cell types present in a tissue with limited *a priori* knowledge. This is a major advance as much of the uncertainty surrounding the status of meiotic sex chromosome inactivation and dosage compensation arises from the fact that expression is traditionally measured in aggregate across entire tissues or body regions. These bulk approaches can mask variability in regulatory patterns across cell types, which is particularly consequential for the gonads which are composed of both somatic and germline cell types.

Here, we combine single-cell RNA-sequencing (scRNA-seq) approaches with a classic sex-ratio distorter in *Teleopsis dalmanni*, the Malaysian stalk-eyed fly. We investigate patterns of sex chromosome regulation and test how meiotic drivers increase their transmission during spermatogenesis and affect the transcriptomic landscape of the testis. *T. dalmanni* has heteromorphic X and Y chromosomes and harbours an X-linked meiotic driver in both wild and captive populations where drive males produce in excess of 90% female offspring [23,44]. The drive X chromosome is thought to have originated around 500 Kya [45] and has multiple impacts on male and female fitness and reproductive traits [21,23,46–52]. The driver influences the outcome of both post- and pre-mating interactions [52,53] but interestingly, drive males have undergone compensatory evolution to match the ejaculate size of standard males and compensate for the destruction of half of their sperm [46,51]. This is most likely via increased testis size in sexually mature flies [54], resulting from larger allocation to the testis primordium at eclosion and a faster growth rate during sexual maturation [47]. Whilst the driver locus remains unknown, recent work has shown that the drive X harbours several inversions relative to the standard X across its entire length, and bulk expression analyses have revealed significant differential expression between driver and standard male testes [17,55]. However, nothing is known about the molecular mechanism of the driver and its consequences for spermatogenesis and gene regulation in the testis more broadly.

First, we produce a comprehensive single-cell atlas of the *T. dalmanni* testis and identify eight major cell types, including somatic supporting cells, germline stem cells (GSC) and spermatogonia, primary spermatocytes, secondary spermatocytes, and spermatids. Next, we characterise the regulation of the standard X chromosome throughout spermatogenesis and provide evidence for a lack of complete meiotic sex chromosome inactivation and complex pattern of dosage compensation across germ cells. Then, by contrasting single-cell expression data between drive and standard males and utilising the time-series nature of scRNA-seq, we test how the meiotic driver biases its transmission to the next generation and influences patterns of sex chromosome regulation. Finally, we highlight genes with perturbed expression as a potential consequence of the disruption of spermatogenesis by the driver.

## Results and discussion

We generated eight individual scRNA-seq datasets from the testes of four standard *T. dalmanni* males and four males carrying the X-linked meiotic driver, referred to as ST and SR respectively. These males were all adult virgins, reproductively mature and reared from egg lays collected on the same day. Standard and drive males only differ in their X chromosome, their autosomes are identical. Following quality control and filtering, we recovered 12,217 cells, in which 12,454 genes were expressed, with 4,609 cells from standard males and 7,608 cells from drive males (S1 Table).

### Single-cell atlas of the *Teleopsis dalmanni* testis

**Fig 1:**
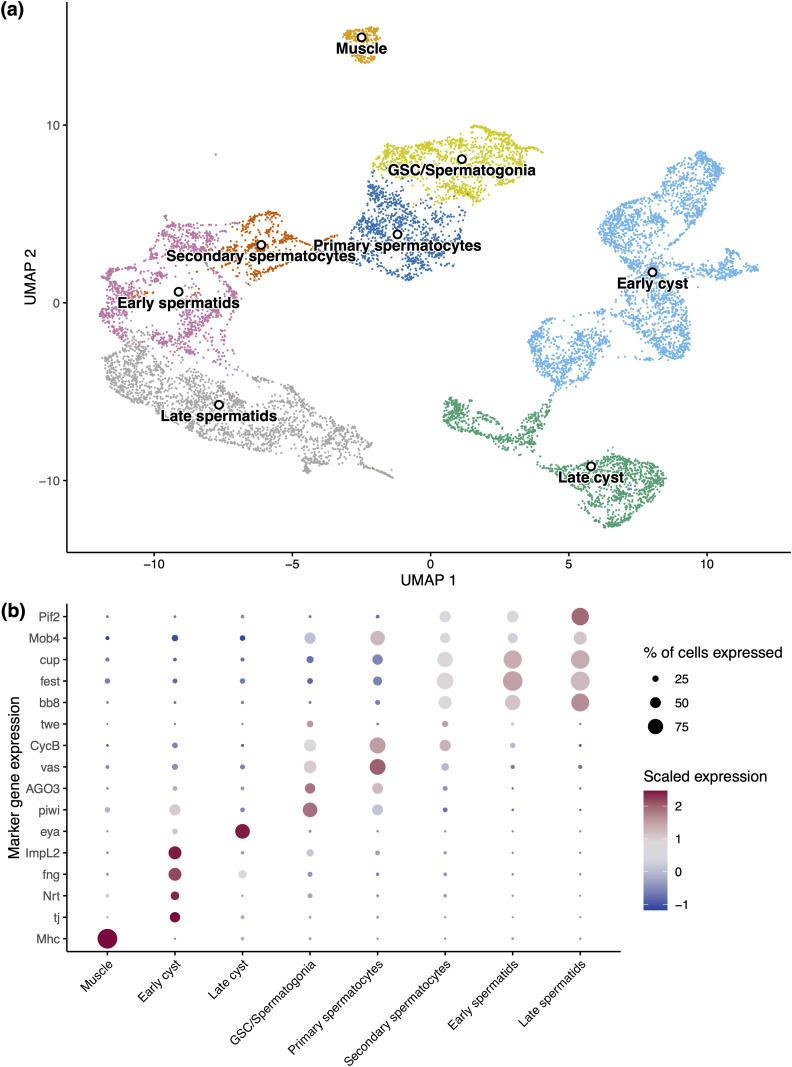
Single-cell atlas of the *Teleopsis dalmanni* testis. (A) Uniform Manifold Approximation and Projection (UMAP) of identified cell types from the testes of standard and drive *Teleopsis dalmanni* males. (B) Dot plot of relative expression of orthologs of key *Drosophila melanogaster* cell-type-specific testis markers. Size of dots indicates the relative number of cells expressing the marker in a cluster and colour indicates the level of expression (blue lowest and red highest). A detailed outline of how we used these markers to distinguish *T. dalmanni* cell types can be found in the S1 Text: Supplementary Results.

Following the clustering of cells via expression patterns, we used orthologs of cell-type-specific markers for *Drosophila melanogaster* testis [56–59] (S2 Table) to identify eight distinct cell types (Fig 1 and S1 Text: Supplementary Results). We identified somatic muscle and two groups of cyst cells which support germline development. We also identified a cluster corresponding to the germline stem cells (GSC) and the spermatogonia they produce. Finally, we were able to distinguish the primary and secondary spermatocytes, which enter meiosis to produce haploid spermatids.

**Fig 2:**
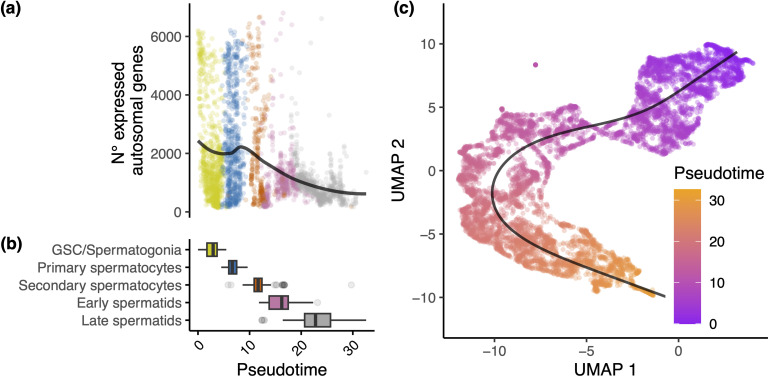
Genome wide expression patterns across *T. dalmanni* spermatogenesis. (A) Number of autosomal genes expressed across spermatogenesis per cell (gene classified as expressed if counts > 1). Data shown for standard (ST) males. Each point represents a cell with colours indicating different cell types as shown on the X axis of panel (b). (B) Boxplot of cell type abundances across pseudotime. (C) UMAP of germline cells, coloured by pseudotime. Plotted line is the principal curve fitted through the centre of the data by Slingshot.

We then used several additional approaches to validate these cell types. First, we used the number of expressed genes to confirm the stages of the germline across spermatogenesis. Previous studies in insect testes have shown that the total number of genes expressed varies significantly across spermatogenesis. Transcriptional activity in the germline peaks before the onset of meiosis in primary spermatocytes, following which transcription dramatically reduces in spermatids [43,58–61]. Consistent with this, we find a clear decrease in the number of expressed autosomal genes over spermatogenesis (Figs 2A, 2B and S1B), supporting our separation of spermatocytes into primary and secondary spermatocytes. Our trajectory analysis, where cells are assigned pseudotimes across a trajectory, further supports this pattern of expression change over developmental time (Fig 2C). Second, we used eukaryotic classifiers of the mitotic cycle stage to corroborate our classification of primary and secondary spermatocytes (S1A Fig and S3 Table and S1 Text: Supplementary Results). Notably, we were unable to use ploidy to distinguish pre- from post-meiotic cell types as proposed by a recent study [62]. We hypothesise several reasons for this and present data urging caution when undertaking this approach with scRNA-seq data (S1 Text: Supplementary Results). Finally, we generated a comprehensive list of markers which are robustly differentially expressed between these cell types for future studies (S4 Table).

### Lack of meiotic sex chromosome inactivation in Teleopsis dalmanni

Next, we characterised patterns of expression across *T. dalmanni* testis cell types in standard males, with a particular focus on the X chromosome. Due to their unique inheritance pattern and characteristics, X chromosomes frequently exhibit sex- and cell-type specific gene regulation compared to the rest of the genome [42,63–67]. For instance, meiotic sex chromosome inactivation acts in many species, inhibiting expression of the X chromosome during the meiotic stages of spermatogenesis [68] and several theories have been suggested to explain its evolution [67]. This regulatory process is thought to have fundamental consequences for the evolution of X-linked coding content and the role of the X in male-specific processes [69]. However, despite its suggested evolutionary importance, the status of meiotic sex chromosome inactivation in insects has remained controversial, with considerable variation reported across studies and species [57,70–79]. In part, this uncertainty arises from the methodological challenges of manually dissecting specific cell populations from testes of multiple individuals, a challenge which scRNA-seq can circumvent [43,57,61].

**Fig 3:**
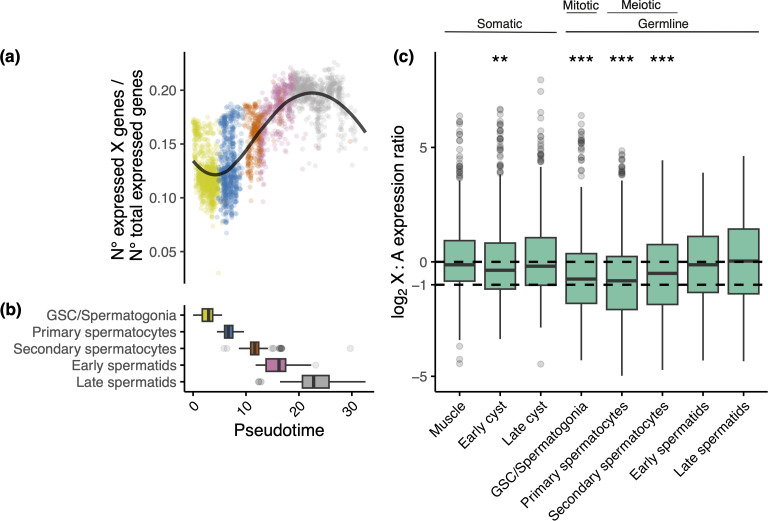
Expression of the X chromosome across *T. dalmanni* spermatogenesis for standard males. (A) Relative number of X-linked genes expressed across spermatogenesis. Data shown for standard (ST) males. For each cell, the number of expressed X-linked genes divided by the number of expressed autosomal genes is shown (gene classified as expressed if counts > 1). Each point represents a cell with colours indicating different cell types as shown on the X axis of panel (b). (B) Boxplot of cell type abundances across pseudotime. The GSC and spermatogonia are mitotic germline cell types whereas spermatocytes are meiotically active. (C) Box plots of the log_2_ ratio of X-linked gene expression to median autosomal expression, measured in counts per million (CPM), across cell types. Data shown for standard (ST) males. Line at 0 represents even expression of autosomal and X-linked genes, consistent with complete dosage compensation and at -1 represents 50% X-linked expression, suggesting incomplete compensation. A two-sided Wilcoxon test was used to determine if log_2_ (X:A) values for each cell type deviate from 0. p < 0.00001 = ***, p < 0.001 = **, p < 0.05 = *.

We used our scRNA-seq data in standard males to test a prominent theory for why meiotic sex chromosome inactivation might evolve in some species but not others. This theory predicts that inactivation evolves to prevent harmful recombination between heteromorphic sex chromosomes [67,80]. If this was the case, we predict an absence of inactivation in species with achiasmatic meiosis, such as the Brachycera suborder of Diptera of which *T. dalmanni* is a member [81], where there is no recombination in males and so no risk of recombination between X and Y chromosomes. As predicted, we find no evidence for a shutdown of the X using several different approaches. First, we find substantial X-linked expression across the five stages of spermatogenesis we identified in *T. dalmanni* (Fig 3A). In fact, the proportion of expressed genes on the X chromosome, relative to the autosomes, is equivalent to or even greater in meiotic and post-meiotic germline cells than in somatic cell types (S2 Fig). Second, following the approach of Elkrewi & Vicoso [73], we classified all somatic and germline cells on the basis of their X:A expression ratio (measured in counts per million), that should reflect inactivation of the X (X:A <=0.33). As expected, we found that no somatic cells were classified as X inactivated. Importantly, we found that virtually none (only 1) of the germline cells were classified as X inactivated (0.0001% of all germline cells) and that this proportion was not significantly different to the somatic cells (p = 1, Chi-square contingency test).

This lack of meiotic sex chromosome inactivation is consistent with recent scRNA-seq data in other achiasmatic Diptera, including *D. melanogaster* [82] (but see Mahadevaraju et al. [57]) and *Drosophila miranda* [43]. Importantly though, the *Teleopsis* X chromosome is not homologous to the *Drosophila* X but instead independently derived from Muller element B [83] and so lends independent support to the theory that inactivation might occur to prevent recombination between the X and Y in males [67,80]. Further anecdotal support comes from recent observations of meiotic sex chromosome inactivation in insects that have been limited to species exhibiting chiasmatic meiosis, where both males and females recombine, including *Anopheles gambiae* [61,84], *Tribolium castaneum* [62,85], *Timema poppense* [72,86] and *Artemia franciscana* [73].

### Status of dosage compensation varies across testis cell types

We next examined patterns of dosage compensation across testis cell types in standard males. Dosage compensation is predicted to evolve on the X chromosome when the X and Y diverge in sequence [87]. This is thought to equalise the expression of sex chromosomes and autosomes in both sexes and mitigate the costs of hemizygous X expression in the heterogametic sex. However, the degree of compensation has been found to vary substantially across species and tissues, particularly between gonadal and somatic tissue [42,72,88–90], but the exact reasons remain unclear [40]. It has been suggested that differences in the magnitude of sexual conflict over optimal expression levels could be responsible [91]. This debate is partly driven by the fact that until recent advances in single-cell approaches [43,57,61,62,82], studies of dosage compensation have measured expression in aggregate across entire tissues or body regions. This masks variability in the degree of compensation across cell types and potentially leads to inaccurate conclusions about the presence or absence of compensation. This is particularly consequential for the testis which is composed of both somatic and germline cell types.

Our scRNA-seq dataset reveals a complex pattern of dosage compensation in the standard *T. dalmanni* testis across spermatogenesis (Fig 3C). Somatic cell types, including muscle and cyst cells, exhibit equal expression of the autosomes and the X chromosome in males, suggesting complete dosage compensation. In contrast, the early stages of spermatogenesis lack dosage compensation, with expression of the X close to half that of the autosomes, whilst equal expression is restored during the later stages (Fig 3C). Notably, in these early stages, X:A expression, as measured in counts per million (CPM), never drops below half and therefore is overall more consistent with lack of dosage compensation than X inactivation. When this pattern is broken down by chromosome, we show that the expression of the two autosomes is constant across testis cell types (S3 Fig). However, the X exhibits a clear relative reduction in expression in the GSC/spermatogonia and primary spermatocytes and then subsequent upregulation in the later stages of spermatogenesis (S3A Fig). We hypothesise that complete dosage compensation is likely facilitated through the sharing of transcripts between haploid sperm cells via cytoplasmic bridges [92]. This is very different to that recently observed using single-cell data in adult *D. miranda* [43] and *D. melanogaster* [82], where there is a progressive shutdown of dosage compensation through spermatogenesis, and in *Anopheles* [61], where dosage compensation is absent in germ cells.

In theory, this variability in X-linked expression relative to the autosomes across the germline could be due to the unique gene content of the T*. dalmanni* X chromosome. Previous work has suggested that the *T. dalmanni* X is highly enriched for testis-specific genes, with almost twice as many as expected based on its size [89]. This masculinisation is not found on the *Drosophila* X [93,94] and so could explain the discrepancies we observe between the two species if male-benefit X-linked genes are disproportionately upregulated in mature sperm in *T. dalmanni*. To test this, we used publicly available RNA-seq data [89] to distinguish *T. dalmanni* genes with testis-specific expression from those broadly expressed across multiple tissues. However, we find that both classes of genes display similar patterns of expression across cell types, with a lack of dosage compensation in early germline cells followed by gradual upregulation across spermatogenesis (S4 Fig). This hints at a chromosome-wide mechanism of X upregulation across spermatogenesis. Our conclusion is further corroborated by the finding that the increase in expression is evenly distributed across the entire chromosome and not limited to a handful of highly expressed genes on the X (S3B Fig). Finally, it is also possible that the upregulation of the X we observe in the latter stages of spermatogenesis to achieve complete dosage compensation is a false signal driven by Y-linked genes that share sequence similarity to the X and are expressed later in spermatogenesis. However, the *T. dalmanni* sex chromosomes are highly diverged with only a handful of Y-linked coding genes being identified to date [83,95]. Given that over 1000 genes are expressed on the X, it is highly unlikely that mismapping of Y-linked reads from this small number of genes could explain the 1) magnitude of the upregulation we observe and 2) the fact it is evenly distributed across the length of the X (100 Mb).

Interestingly, while we found differences in patterns of dosage compensation in the germline between *Drosophila* and *T. dalmanni*, orthologs of key components and accessories of the dosage compensation complex (DCC) that operates in the soma of *Drosophila* [96] show similar expression between the two species [43,82]. As in *Drosophila*, we find that these genes exhibit a gradual reduction in expression across stalk-eyed fly spermatogenesis (S5 Fig). Male-specific lethal (MSL) gene recognition sites, key parts of the *Drosophila* compensation complex, have yet to be identified in *Teleopsis* and so we are unable to test directly whether differential gene activity correlates with physical proximity to chromatin entry sites. However, there is currently mixed evidence for the role of the DCC complex in regulating dosage compensation more broadly in the insect germline [43,62,82,97], particularly as components of the complex do not localise to the nucleus [82] or the X in the *D. melanogaster* male germline [98]. Given the lack of concordance we find between expression of the whole X and components of the DCC, our results further support a non-canonical mechanism of dosage compensation in insect testes.

### Impacts of meiotic drive on the cellular landscape of the testis

The mechanisms by which drivers bias their transmission to gametes have only been studied in a handful of species [26]. However, they appear to operate through two main approaches, either by killing gametes directly or halting their maturation. Meiotic-acting drivers disrupt proper segregation at meiosis, such as the *Paris* driver in *Drosophila simulans* which causes improper segregation of the Y in anaphase II [99,100]. In contrast, post-meiotic drivers disrupt motility of sperm or poison them. For instance, the *Winters* driver in *D. simulans* leads to a defect in nuclear condensation of Y sperm [101]. To test which of these mechanisms is operating in *T. dalmanni* we compared the cellular composition of the testis across spermatogenesis between drive and standard males.

In *T. dalmanni,* sperm are formed in bundles from cyst cells, eventually composed of 128 mature germ cells housed in two cyst cells [44]. If the driver acts by killing Y bearing sperm directly, we expect to see a relative depletion in the number of germ cells compared to supporting cells found in later stages of spermatogenesis in drive males. This is because if Y bearing sperm were killed or not formed during meiosis, we would expect bundles to contain less than 128 germ cells, and thus a relatively smaller number of germ cells relative to cyst cells in drive compared to standard males. However, if the driver prevents Y bearing sperm from fully maturing, we expect to see no difference or even a relative increase in cell numbers towards the end of spermatogenesis. This is because immobilised or improperly elongated Y sperm (or O sperm if improper segregation at meiosis) may be unable to migrate to the seminal vesicle and so temporarily accumulate in the testis.

First, we did not confidently observe any cell types unique to males carrying the meiotic driver relative to standard males (S1 Table). Secondly, we see no significant effect of meiotic drive on relative cell type abundance when comparing the relative size of the cyst to the germline across males (S1 Table, p = 0.13). This lack of a clear difference in the relative number of cells progressing through spermatogenesis between standard and drive males suggests there is no sudden sperm cull, but instead that the driver causes incomplete spermatid maturation. Therefore, once Y bearing sperm are immobilised, they may simply build up in the distal end of the testis before being eliminated by standard cellular programs. Indeed, we do observe a non-significant relative enrichment for post-meiotic germ cells in drive individuals (S1 Table, p = 0.06).

Previous research has shown that the presence of the meiotic driver has selected for increased testis size in drive males, attributed to compensatory evolution for the loss of Y bearing sperm [47]. This would be achieved by an equivalent increase in the size of supporting tissues, including cyst cells, and the subsequent number of X and Y-bearing germ cells produced overall. Consistent with this, we did recover a larger number of cells from drive relative to standard males, potentially due to the larger amount of tissue available from drive testes (S1 Table).

Together, our findings suggest the drive X chromosome influences development by preventing full elongation of presumably Y-bearing spermatids. This is consistent with previous cytological work in *T. dalmanni* where sperm of drive males reach the latter stages of spermatogenesis, but, just before individualization, sperm heads either deteriorate before leaving the bundle or appear overextended [44,102]. Together, this pattern is analogous to the *Segregation Distorter* (SD) male meiotic drive system in *D. melanogaster*, where the driver operates post-meiotically to prevent sperm maturation [13] and consistent with cytological studies in closely related *Teleopsis whitei* where drive males had more nonelongated sperm heads than standard males [103].

### Impacts of meiotic drive on the transcriptional landscape of the testis and sex chromosome regulation

In theory, we might expect meiotic drivers to perturb broad patterns of sex chromosome regulation through direct and indirect processes. Meiotic drivers are frequently housed by inversions [19,104,105] and the *T. dalmanni* driver is no exception. Although it’s location on the X chromosome is unknown, the drive X is characterised by a series of large inversions across its entire length [17,102]. A consequence of these inversions is reduced recombination for both the standard and drive X chromosomes, leading to high sequence divergence between X types and low diversity within the drive X [17]. In theory, this permits compensatory evolution via the linkage of advantageous alleles to the driver to mitigate reductions in fertility. Furthermore, inversions may directly disrupt cis-regulation by physically shuffling promoters and enhancers within a chromosome [106], potentially leading to misexpression of key genes involved in other traits. Therefore, using our scRNA-seq data, we tested the extent to which the meiotic driver perturbs fundamental patterns of sex chromosome regulation and gene expression more broadly across spermatogenesis.

**Fig 4:**
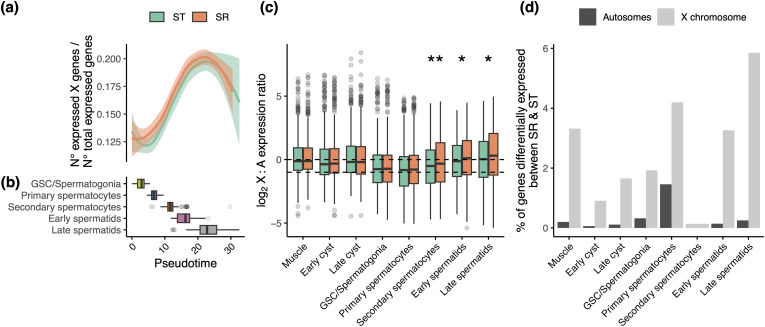
Conservation of X-linked regulation in the germline of drive males. (A) Loess curves fit to the relative number of X-linked genes expressed across spermatogenesis for standard (ST) and drive (SR) male cell types separately. For each cell, the number of detected X-linked genes divided by the number of expressed autosomal genes (gene classified as expressed if counts > 1) is shown. Filled area is the standard deviation. (B) Boxplot of cell type abundances across pseudotime. (C) Box plots of the log_2_ ratio of X-linked gene expression to median autosomal expression, measured in counts per million (CPM), across cell types in standard (ST) and drive (SR) males. Line at 0 represents even expression of autosomal and X-linked genes and at -1 represents 50% X-linked expression and complete lack of dosage compensation. A two-sided Wilcoxon test was used to determine if values for each cell type varied between ST and SR individuals. p < 0.05 = * (D) Percentage of expressed autosomal (dark grey) or X-linked (light grey) genes in each cell type that were differentially expressed between ST and SR individuals.

First, we find no evidence for meiotic sex chromosome inactivation (Figs 4A, 4B and S6) and similar patterns of dosage compensation on the drive X to standard males (Fig 4C). The only exception is a marginal increase in relative X-wide expression from secondary spermatocytes onwards in drive relative to standard males (p < 0.05). Together, these patterns suggest that there is no widespread dysregulation of the X chromosome across spermatogenesis because of meiotic drive.

Strikingly, we observe only a limited number of genes that are differentially expressed between standard and driver males across either the autosomes or X chromosome (Figs 4D and S7, S5 and S6 Tables) (120 autosomal and 126 X-linked genes in total), although this may be somewhat attributed to lower cell counts for some cell types [107]. These differentially expressed genes are disproportionately located on the X chromosome across cell types (p < 1x10^-5^) (Fig 4D and S5, S6 and S7 Tables), evenly distributed across the entire X and not localised to a specific region (S8 Fig). The differential expression we observe, particularly on the X, is significantly more than we would expect based on variability in expression between standard males alone (S9 Fig). There was no significant relationship between rates of coding sequence evolution and differential expression (S1 Text: Supplementary Methods and S8 Table). Notably, we found a significant and positive association across all genes between the magnitude of differential expression and differences in coverage (a proxy for copy number variation) between standard and drive males (p = 0.024, ρ = 0.027 across all genes, p = 0.0008, ρ = 0.6 across genes with significant differential expression and coverage) (S10 Fig), consistent with previous research [17].

Next, we identified genes with significantly differential trajectories between standard and drive spermatogenesis (S9 Table and S11 Fig). Trajectory analyses allow us to test whether genes are differentially regulated across development, rather than at distinct, self-assigned timepoints that include cells spanning developmental states. Briefly, we assigned pseudotime points to each germ cell and fitted a Generalised Additive Model (GAM) for standard and drive cells independently for expression of each gene. To ensure high confidence in the identified trajectories, genes with no association between pseudotime and expression were removed (n = 400) leaving 5,985 genes for this analysis. Using the top 500 genes with differential trajectories between standard and drive spermatogenesis, ranked by Wald statistic, enrichment was seen for gene ontology terms including cilium, sperm motility and cell projection assembly (S10 Table).

Finally, we sought to identify candidates for functional aspects of the driver. Disruption to spermatogenesis by the driver is likely to perturb normal processes of sperm development leading to differential expression as a by-product. Therefore, we combined our analyses of differential expression and trajectories with our copy number analyses to pinpoint potential mechanisms by which the driver operates. This is because meiotic drivers are frequently characterised by expansions in copy number [108–110]. Previous research identified seven genes that exhibit both differential expression and differential genomic coverage between whole testes of drive and standard males [17,55]. Of these genes, five were not differentially expressed in our dataset (S11 Table). This discrepancy is potentially an outcome of measuring differential expression from bulk approaches, which represent an average of expression across entire populations of distinct cell types and can lead to false inferences of regulatory variation [111–113]. *mcm10*, a protein coding gene with a predicted role in DNA replication and heterochromatic silencing, was drive-biased in primary spermatocytes and late spermatids. Importantly, we found significant drive-biased expression of a copy of *JASPer* in primary spermatocytes (S12 Table)*.* This gene has undergone a significant expansion in copy number on the drive X [17] and has a role in the maintenance of euchromatin and regulation of TE expression.

Using our data, we were able to identify several new candidates (S7 and S11 Figs and S6 and S9 Tables). Notably, we identified differential expression of *tsr*, *Drosophila* mutants of which are unable to perform proper cytokinesis at meiosis I and II [114]. This gene is X-linked, has significantly lower expression in spermatocytes of drive individuals, exhibits a differential trajectory but shows significantly reduced coverage in drive males, indicative of lower copy number (S6 Table). Similarly, *Grip75* is required for tethering of microtubules, and *Drosophila* mutants are sterile with defects in meiosis and sperm motility [115]. Consistent with this phenotype, *Grip75* is X-linked and expressed at significantly lower levels in *T. dalmanni* drive individuals, particularly in the later stages of spermatogenesis, but without a coverage bias. *Dnaaf4*, a gene vital for ciliary motility [116] exhibits drive-biased expression in primary spermatocytes and GSC/spermatogonia, as well as a differential trajectory and strong coverage bias towards drive individuals. Finally, *Ced-12* is required for apoptotic cell clearance [117] with *Drosophila* mutants showing significantly increased spermatogonia volume [118]. *Ced-12* is X-linked and downregulated in the later stages of spermatogenesis in drive individuals and with unbiased coverage. With strong selection pressure for increased testis size acting in drive males [47], this shift in regulation of testis growth is a clear potential mechanism to mitigate the loss of Y-bearing sperm.

## Conclusion

In conclusion, we provide a comprehensive profile of the cellular and transcriptional landscape of the testis of the stalk-eyed fly. Specifically, we show limited evidence for meiotic sex chromosome inactivation and unique patterns of dosage compensation across spermatogenesis, relative to both other dipterans and insects in general. Additionally, we highlight genes with perturbed expression as a potential consequence of the disruption of spermatogenesis by the X-linked meiotic driver in this species. Together, our results suggest that this driver likely acts by interfering with proper sperm development, rather than directly killing gametes.

## Methods

### Reference genome and mitochondrial genome assembly

The reference genome for *Teleopsis dalmanni* [119–121] consists of two autosomes and an X chromosome. However, it lacks a mitochondrial sequence. We therefore assembled a mitochondrial genome using publicly available PacBio Hifi reads generated from pooled *T. dalmanni* larvae [120] and MitoHifi v3.01 [122]. Specifically, we used raw Hifi reads as input, the rust fly (*Loxocera sinicia*) mitochondrial genome as a reference, and MitoFinder to annotate the genome, to produce a circularised assembly 20,708 bp in length containing 37 genes. This *T. dalmanni* mitochondrial reference is available [123]. The reference genome lacks a Y chromosome, but it is highly diverged from the X and contains only a handful of coding genes [17,83,95].

### Sample collection

Flies were reared at University College London from wild-caught populations originating from the Gombak Valley, Malaysia [49]. All flies and larvae were incubated and reared at 25°C and fed on a diet of sweetcorn. To ensure known genotypes of samples, a homozygous drive (SR) population is maintained through a series of crosses as previously described [44,49]. Standard (ST) and drive (SR) males only differ in their X chromosome, their autosomes are identical, as the breeding regime involves crosses between stock populations containing the two chromosomal types, which means they do not differ in their Y chromosome or autosomes. Eight adult males, four standard (ST) and four with drive (SR), were sacrificed before the dissection of both testes in iced phosphate-buffered saline (PBS). These adults were all virgins, reproductively mature and reared from egg lays collected on the same day.

### Tissue collection, dissociation and single-cell sequencing

Testes pairs were individually dissociated by incubation in a collagenase-TrypLE lysis solution (10mg/ml collagenase in 10X TrypLE) at 37.5°C for one hour with three sets of mechanical dissociation by triturations of wide then narrow bore Pasteur pipettes. Digestion was inhibited by the addition of iced Schneider’s Serum. The solution was then gently triturated with a narrow-bore Pasteur pipette before filtering through a 35μm filter pre-rinsed with Schneider’s Serum. The sample was then spun in a swing bucket centrifuge for 5 minutes at 1000xg and 4°C. The supernatant was removed and the pellet resuspended in 50μl of iced PBS with gentle pipetting of a wide-bore pipette. To count cells, 10μl of the suspension was combined with 10μl of trypan blue and placed onto a humidified haemocytometer plate before counting in triplicate.

10X Genomics Chromium transcriptome libraries were generated at the NERC Environmental Omics Facility (NEOF) Liverpool before sequencing with Illumina NovaSeq using S2 chemistry, aiming for recovery of ~10,000 cells per sample and ~20,000 reads per cell. Raw scRNA-seq data for eight males is available [124].

### Single-cell RNA-seq data processing

Sequencing data for each sample was processed using Cell Ranger v7.2.0 [125]. First, a custom reference genome was built with the *T. dalmanni* reference genome using mkref. Using cellrangers count function, fastq reads were then aligned against the custom index and counted, creating gene-by-cell count matrices. Data filtering and downstream analyses were performed using Seurat v5.1.0 [126,127] in R v4.3.2 [128]. Cells in each sample were removed from the analysis if they expressed less than 200 features and more than 20% mitochondrial expression (S12 Fig). 21% of SR cells and 19% of ST cells fail the 20% filter and were removed. Count data for each sample was also filtered by only keeping genes with expression (counts > 1) in at least three cells. We used DoubletFinder v2.0.4 [129] in R [128] with default parameters to identify and remove doublets. It is possible that our dataset contains some cells with multiple nuclei because they have yet to fully complete cell individualisation post meiosis at the point we dissected the testes. However, we expect the nuclei in these multinucleated cells to produce similar transcriptomes and so we do not expect this to bias our results. If in fact their expression profiles are dissimilar then we expect them to be removed via our doublet filtering step.

The filtered dataset consisted of 12,217 cells across the eight samples, expressing 12,454 genes, with 7,608 cells from drive individuals and 4,609 cells from standard individuals (S1 Table). Seurat objects from all eight samples were then integrated post-filtering using the ‘SCTransform’ function [130].

### Cell-type identification

We conducted cell type clustering and identification on the combined dataset of standard and drive males. After running a PCA on the integrated Seurat object, we used the ElbowPlot function to identify how many PCs were necessary to describe a significant amount of variation. Subsequently, a nearest neighbour graph was created using FindNeighbors, and clusters at varying resolutions identified with FindClusters. From this, an appropriate resolution for the number of clusters was determined using the clustree package v0.5.1 [131], giving a final cluster number of 16.

Using a series of cell-type-specific markers for *Drosophila melanogaster* testis (S2 Table) [56,57,59], clusters were assigned into biological groupings. Orthology between *T. dalmanni* and *D. melanogaster* reference genome (dm6) was established using OrthoFinder v2.5.5 with default parameters [132] giving a total of 9,883 reciprocal orthologs. Distinguishing cell populations in non-model organisms relies primarily on databases of marker genes from model species, which are often distantly related. Our comparison between *T. dalmanni* and *D. melanogaster* (a divergence time of ~150 MY) is within the range of species pairs previously employed to identify cell types using orthologous marker genes in recent single-cell RNA-seq studies [62,133,134].

To further validate cell types, we used information on the number of features expressed and classifiers of the mitotic cycle stage (S3 Table). Finally, we cleaned the data by removing clusters that (a) were predominantly represented by only a few samples (a cluster must have at least three samples from a treatment representing >5% of the cells, weighted by total cell number for each sample) or (b) had no clear biological classification (S13 Fig and S1 Text: Supplementary Results). After identifying cell types, new markers were identified based on differential expression using FindAllMarkers from the Seurat package.

### Dosage compensation analysis

We tested for dosage compensation separately for standard and drive samples. For each sample and cell type, raw counts were aggregated using a pseudobulk approach with scuttle v1.14.0 [135,136]. Specifically, the counts of all cells belonging to a cell type were summed for each gene across the genome. Using a pseudobulk approach instead of the expression of each cell reflects that the sample is the biological replicate and not the cell itself [137] and has been shown to reduce false positives in scRNA-seq analyses [136]. Aggregated counts were then normalised for library size using normLibSizes from edgeR [138]. Subsequently, genes for each cell type were filtered in two ways. First, genes were kept if they were expressed in>= 5% of cells (> 1 count). Second, genes had to have a pseudobulk log_2_(CPM) (counts per million) > 2 in more than half of standard or drive males. Dosage was measured as the ratio of CPM values for X-linked genes to the median autosomal CPM value. In each cell type, a non-parametric two-sided Wilcoxon test was used to test for deviations of log_2_(X:A) from 0 in standard males, with μ set to 0, and differences in log_2_(X:A) between standard and drive males.

### Differential cell type abundance analysis

To test for differences in cell type abundance between standard and drive males, a series of binomial models were fit comparing cell counts of germline to cyst, early cyst to late cyst, and pre-meiotic germline to post-meiotic germline. All models were run using glmer from lme4 [139] in R [128] with sample as a random effect (cell type~treatment + (1|sample)).

### Differential gene expression analysis

Following the same pseudobulk approach used for the dosage compensation analysis, differential expression was analysed between drive and standard males. A quasi-likelihood (QL) approach from EdgeR v4.0.16 [138] was used to identify differentially expressed genes in each cell type (|log_2_(fold-change)| > 1 and FDR < 0.05). Enrichment of the number of differentially expressed genes across chromosomes and cell types was modelled with a generalised linear model of family ‘binomial’ where genes were classified as biased or unbiased and regressed against cell type and chromosome (Significant ~ chromosome * cell type). Nested models were then compared using chi-squared in anova.glm from the R ‘stats’ package [128].

### Coverage variation analysis

An additional dataset of paired-end short read Illumina DNA resequencing for 77 wild individuals (ST_n_ = 50, SR_n_ = 27) was used for estimation of coverage variation as a proxy for differences in copy number differences between standard and drive males [140]. Reads were trimmed with Cutadapt v1.2.1 [141] and Sickle v1.2 [142]. Trimmed reads were then aligned with Bowtie v2.4.1 [143] and PCR duplicates removed using MarkDuplicates from GATK v2.27.5 [144], before sorting of resultant BAM files with samtools v1.21 [145]. Coverage differences were then estimated using the exact test in edgeR v4.2.2 [138] on the gene counts matrix generated by featureCounts [146].

### Trajectory analysis

To identify genes with differential trajectories between standard (ST) and drive (SR) males across spermatogenesis, data was subset to include only germline cells (GSC/spermatogonia, primary and secondary spermatocytes, and spermatids). The Seurat object was then converted into a SingleCellExperiment class for downstream analysis using tradeSeq v1.18.0 [147] and slingshot v2.12.0 [148]. First, pseudotimes were assigned to each cell within the germline. Then a negative binomial generalized additive model (NB-GAM) with 8 knots was fit to each gene for ST and SR individuals separately. Genes were kept if they were expressed in at least 10% of either ST or SR cells with 2 or more counts [147], and if their expression was significantly associated with pseudotime in either ST or SR cells (*p*_*fdr*_ < 1 x 10^-6^ & log_2_ fold-change > 1). GAM smoothers were then compared between ST and SR cells to identify genes with significantly different trajectories using the conditionTest function. Genes were classed as significant if false discovery rate *p*_*fdr*_ < 1 x 10^-6^ & log_2_ fold-change > 1.

### Gene Ontology enrichment analysis

A gene ontology term enrichment of the top 500 genes, ordered by Wald statistic, with significant differential trajectories was performed using the clusterProfiler package v4.12.2 [149]. The background gene set used was the genes that were previously identified as having a significant association with pseudotime. The org.Dm.e.g.,db v3.19.1 [150] package for *D. melanogaster* was used as a reference database.

## Supporting information

S1 Text This document contains Supplementary Methods and Results. (DOCX)

S1 Fig Dot plot of all markers to define cell types & phase UMAP figure. (A) Uniform Manifold Approximation and Projection (UMAP) of cells classified by mitotic stage marker expression as a proxy for cell cycle stage. G1: Gap 1, S: DNA synthesis, G2M: Gap 2/Mitosis (B) Boxplots of number of autosomal genes expressed across all cell types (gene classified as expressed if counts > 1). (C) Dot plot of relative expression of orthologs of *Drosophila melanogaster* cell-type-specific testis markers. Size of dots indicates the relative number of cells expressing the marker in a cluster and colour indicates the level of expression (blue lowest and red highest).
(TIFF)

S2 Fig Genome wide expression patterns across *T. dalmanni* spermatogenesis. Violin plot showing the relative number of X-linked genes expressed across *T. dalmanni* cell types in standard (ST) males. A two-sided Wilcoxon test was used to determine if values in (A) differed across the stages of spermatogenesis where p < 0.00001 = ***, p < 0.001 = **, p < 0.05 = *.
(PDF)

S3 Fig Expression of the X chromosome and autosomes across cell types. (A) Expression values of genes in standard (ST) males from chromosome 1, 2 and the X chromosome across cell types. Expression values were compared in a pairwise manner between chromosomes with a two-sided Wilcoxon test. p < 0.00001 = ***, p < 0.001 = **, p < 0.05 = *. (B) Distribution of gene expression across the genome in each cell type. Expression is measured as the average log_2_(CPM) across ST males for each cell type, utilising a pseudobulk approach.
(TIFF)

S4 Fig Dosage compensation of testis-specific and universally expressed genes. Box plots of the log_2_ ratio of X-linked gene expression to median autosomal expression, measured in counts per million (CPM), across cell types. A two-sided Wilcoxon test was used to determine if values for each cell type and class of tissue-specificity differed from 0. p < 0.00001 = ***, p < 0.001 = **, p < 0.05 = *.
(TIFF)

S5 Fig Expression of orthologs of the *Drosophila* dosage compensation complex. Dot plot of scaled expression of *T. dalmanni* orthologs of components and accessory genes of the dosage compensation complex (DCC) in *Drosophila*. Colour signifies scaled expression and dot size represents the percentage of cells expressing the specific gene.
(TIFF)

S6 Fig Comparing expression of the X chromosome across cell types in standard (ST) and drive (SR) males. Box plot showing the relative number of X-linked genes expressed across cell types in standard (ST) and drive (SR) males. A two-sided Wilcoxon test was used to determine if the proportion of expressed X-linked genes for each cell type differed between ST and SR cells. p < 0.00001 = ***, p < 0.001 = **, p < 0.05 = *.
(PDF)

S7 Fig Differential gene expression across cell types. Volcano plots of differentially expressed genes in each cell type for (a) the X chromosome and (B) the autosomes with log_2_ fold-change on X axis and False Discovery Rate (FDR) adjusted p-value on the Y axis. Blue points are unbiased genes and green and orange dots are drive (SR-) and standard (ST-) biased genes respectively (FDR < 0.05 and absolute log_2_(fold-change) > 1). Labelled points are the top 8 significant genes per cell type (ordered by FDR) with *Drosophila* orthologs (Note that not all cell types have 8 genes matching this criteria).
(TIFF)

S8 Fig Spatial patterns of differential gene expression across cell types. Dot plots of differentially expressed genes in each cell type across the genome with chromosomal position on X axis and log_2_ fold-change on Y axis. Blue points are unbiased genes and green and orange dots are drive (SR-) and standard (ST-) biased genes respectively (FDR < 0.05 and absolute log_2_(fold-change) > 1).
(TIFF)

S9 Fig Pseudo-replicate validation of X-biased differential expression. To validate enrichment of differential expression to the X, pseudo-samples were generated from the pool of ST and SR cells. For ST vs ST comparisons, two sets of four pseudo-samples of 100 cells were produced. These cells were sampled with replacement from the pool of all ST cells. The percentage of genes differentially expressed between each ST group was then calculated for the X and autosomes. The same approach was then applied to compare pseudo-samples between ST and SR, creating four pseudo-samples for ST and four for SR. A distribution of the % of differentially expressed genes in each comparison was generated through 1000 repeats. A Wilcoxon test was used to determine whether the proportion of genes differentially expressed in ST vs SR differed to ST vs ST.
(TIFF)

S10 Fig Coverage variation. (A) Scatter plot of average log_2_ fold-change of expression against log_2_ fold-change in coverage between ST and SR. Relationship determined by a Spearman’s rank. (b) Boxplot comparing genes that exhibit significant (p < 0.05) differential expression and differential coverage (n = 28). For this set of genes, a Wilcoxon test was used to determine whether coverage varied between ST- and SR-biased genes.
(TIFF)

S11 Fig Differential trajectories of selected genes across spermatogenesis. Differential trajectories for cells assigned to germline cell types (GSC/spermatogonia, primary and secondary spermatocytes, and spermatids). Plotted are genes identified as those that are both differentially expressed and with differential trajectories between standard (ST) and drive (SR) individuals (p-value < 0.05 & log_2_ fold-change > 2). Genes are then ordered by descending Wald stat from the condition test for identifying differential trajectories with the top 20 shown. D.mel refers to genes with *Drosophila melanogaster* orthologs, and Ref gene are those without.
(TIFF)

S12 Fig Mitochondrial expression and number of expressed features. UMAP of raw data before filtering for mitochondrial expression or selection of cell types (a and b) and number of expressed features (c and d) for ST and SR samples. Colour intensity in (A) and (B) represents the level of mitochondrial expression measured as the percentage of transcripts in each cell mapping to the mitochondrial genome. Colour intensity in (c) and (d) represents the number of expressed features in each cell. 21% of SR cells and 19% of ST cells failed the 20% mitochondrial expression filter and were removed. Most cells that fail the 20% filter fall into a single cluster which is represented by both standard and drive cells. Notably, cells in this cluster also express very few genes (C and D), which is highly symptomatic of dying cells. Importantly, this cluster is a similar size between ST and SR. Therefore, we do not think it is a biologically real cluster of cells dying due to driver action but is instead an artifact where all dying cells across the testes have clustered together because of their unique expression profile (e.g., high mitochondrial expression and low no. of features).
(TIFF)

S13 Fig Unfiltered single-cell data set. Single-cell data set before removal of clusters with no clear biological classification or that were predominantly represented by a single sample. (A) UMAP of identified cell types from unfiltered single-cell data sets for ST and SR samples. (B) Boxplots of number of genes expressed across cell types (gene classified as expressed if counts > 1).
(PDF)

S14 Fig Using ploidy to distinguish cell types in standard (ST) samples. (A) Percentage of sites per cell that are homozygous (all reads matching either reference or alternate at a site). Data only shown for standard (ST) male diploid cell types (cyst, muscle, GSC/spermatogonia, and primary spermatocytes) No depth or minimum number of site thresholds were set for calling the ploidy of each cell. (B) Number of cells classified as haploid or diploid for each cell type following the filtering in (A). (C) and (D) are the same as (A) and (B) however a threshold of being genotyped at>= 10 sites per cell with depth of>= two for calling homozygous or four for heterozygous (two mapping to both ref and alt).
(PDF)

S1 Table Cell numbers. (XLSX)

S2 Table Marker genes [151–172]. (XLSX)

S3 Table Cell cycle markers. (XLSX)

S4 Table Novel markers. Novel marker genes identified using FindAllMarkers from Seurat. In brief, for each cluster, differentially expressed genes were identified between the cluster and all other clusters in the dataset.
(XLSX)

S5 Table Differential expression. (XLSX)

S6 Table Differentially expressed genes. Differentially expressed genes. M = Muscle, EC = Early cyst, LC = Late cyst, G = GSC/Spermatogonia, PS = Primary spermatocytes, SS = Secondary spermatocytes, ST = Spermatids.
(XLSX)

S7 Table Differential gene expression enrichment model. (XLSX)

S8 Table dN/dS estimates. (XLSX)

S9 Table Differential trajectories. Top 500 genes with differential trajectories as identified by tradeSeq. Degrees of freedom are determined by the complexity of the fitted trajectory, with a maximum of 8. This value was determined through optimisation of the bias-variance trade-off using evaluateK from the tradeSeq package.
(XLSX)

S10 Table Trajectory gene ontology terms. BP = Biological Process, MF = Molecular Function, CC = Cellular Component
(XLSX)

S11 Table Reinhardt et al candidates [17]. Expression and coverage of the seven candidate genes identified in Reinhardt et al 2023 Genome Biology and Evolution in our dataset. [17]
(XLSX)

S12 Table JASPer paralogs. Expression and coverage of the JASPer paralogs in our dataset.
(XLSX)

S13 Table Inbreeding values. (XLSX)
