# Supplementary material for: Single-cell consequences of X-linked meiotic drive in stalk-eyed flies
Source: PLoS Genet. 2025 Sep 18;21(9):e1011816. doi: 10.1371/journal.pgen.1011816 (PMC12445520; doi:10.1371/journal.pgen.1011816)
Supplement: S1 Text — (DOCX) [file pgen.1011816.s001.docx]

# **S1 Text**

## **SUPPLEMENTARY METHODS**

***Alignment, SNP calling and heterozygosity calculations***

DNA for nine *T. dalmanni* individuals was extracted using standard approaches. Illumina DNA-seq libraries were generated at the NERC Environmental Omics Facility (NEOF) Liverpool before sequencing with Illumina NovaSeq using S4 chemistry, aiming for a coverage of 30X.

FASTQ files were quality trimmed and aligned to the indexed *T. dalmanni* reference genome (https://doi.org/10.5061/dryad.j6q573nqw) using the BWA-MEM algorithm implemented in BWA v0.7.17 [1]. BAM files were sorted and indexed using Samtools v1.11 [2]. Read group information was added to the BAM files using Picard tools v2.27.5 (http://broadinstitute.github.io/picard) and PCR duplicates removed using Picard tools MarkDuplicates. Next, variant calling was performed using GATK HaplotypeCaller v4.3.0 [3] to generate GVCF files and remove reads with a mapping quality < 20 and base quality score < 20. GVCFs were merged into a single GVCF using GATK CombineGVCFs and genotypes called using GATK GenotypeGVCFs. Variant filtering was performed using BCFtools v1.11[4]. Genotypes of sites were set to missing (.) if they met one of the following parameters: Depth (DP) < 5 or > 98, genotype quality (GQ) < 50, or SNP quality (QUAL) <= 50. Additionally, only biallelic SNPs were kept. Then, BCFtools view was used to extract only the autosomes (Chr1; Chr2) and the extracted vcf file was indexed using Tabix (htslib) v1.13[5]. Levels of heterozygosity and the inbreeding coefficient (F) per individual were calculated using VCFtools (-het) v0.1.17 [2].

***dN/dS estimates***

Reciprocal orthologs were identified for gene sequences between *Teleopsis dalmanni*, *Drosophila melanogaster, Episyrphus balteatus* and *Hermetia illucens* using OrthoFinder v2.5.5 with default parameters [6]. Orthologous nucleotide sequences were aligned using PRANK v.170427 [7] and then masked with SWAMP v1.0 [8] using a mask of 7 mismatches in a 12 base window followed by a mask of 4 mismatches in a 7 base window. The branch model in PAML v4.8 [9] was used to estimate dN/dS values in *Teleopsis dalmanni*, removing genes with dS >2 to account for mutational saturation. For genes classed as SR-, ST-, or unbiased in expression (bias in at least one cell type), mean dS and mean dN were calculated by summing the number of nonsynonymous sites (N), synonymous sites (S), nonsynonymous changes (DN), and synonymous changes (DS). $dN=\frac{\sum_{g=1}^{n} DN_{g}}{\sum_{g=1}^{n} N_{g}} dS=\frac{\sum_{g=1}^{n} DS_{g}}{\sum_{g=1}^{n} S_{g}}$, where n is the number of genes within a expression bias class. This approach overcomes infinite dN/dS values due to zero or very low dS values. Bootstrapping with 10000 replicates generated confidence intervals and 2-sided p-values for comparisons between gene classes.

## **SUPPLEMENTARY RESULTS**

## ***Expression of marker genes across cell types in Teleopsis dalmanni testis***

First, we used a series of known *Drosophila melanogaster* cell-type-specific testis markers [10-13] to identify *T. dalmanni* cell types (Figures 1 & S1c, Table S2). We used the germ-cell-specific marker *vasa* to separate the germline from somatic tissues [14]. Somatic tissues were split using *Mhc* to identify muscle cells [15], *eya* expression to identify cyst cells associated with post-mitotic germline cells [16], and *Impl2*, *fng*, *tj* and *Nrt* to identify cyst cells associated with the mitotic germline [17]. To then separate the germline stem cells (GSC) and spermatogonia from meiotic phase cells, *bb8* was used, a key gene in mitochondrial derivative development, which is expressed from spermatocytes onwards [18]. The lack of *twe* expression was used to split spermatocytes from spermatids, with *twe* expression peaking in spermatocytes and not detected in spermatids [19]. The presence of *cup* genes could also validate spermatids as whilst the post-meiotic germline of *Drosophila* is transcriptionally nearly inactive, *cup* and *comet* genes are expressed [20]. To then separate primary from secondary spermatocytes, we used expression of *CycB*, a core G2/M cell cycle component, whose expression peaks at the meiosis I transition between primary and secondary spermatocytes, and *Fest*, a regulator of *CycB*, whose expression begins in primary spermatocytes and extends into late-stage germ cells [21]. Late spermatids were identified using expression of *Mob4* [21] and *Pif2* [10], which show a 0.5 log_2_ fold-change increase and 1 log_2_ fold-change increase in late spermatids respectively, both with p < 1x10-20.

These key markers, used to define cell types, were highly expressed in our dataset and shown in Figure 1c. However, there is a wider set of markers in the literature that we used to corroborate our findings, shown in Figure S1c and Table S2, but some of these genes were lowly expressed in our dataset and so less reliable in defining cell types.

We then used a large set of eukaryotic mitotic cycle stage classifiers, from the Harvard Chan School: Bioinformatics Core (https://github.com/hbc/tinyatlas/blob/master/cell_cycle/Drosophila_melanogaster.csv), (Figure S1a, Table S3) to refine the stages of spermatogenesis. Expression of G2M markers was enriched in the germ-line stem cells through to primary spermatocytes in line with the DNA damage checkpoints of mitotic and first meiotic divisions following DNA synthesis [23].

### ***Using ploidy to distinguish cell types across spermatogenesis***

A recent study proposed using SNP-based haploid/diploid phasing to distinguish pre- from post-meiotic cell types [23]. We expect the GSC/spermatogonia and primary spermatocytes to be diploid and secondary spermatocytes and spermatids to be haploid. Therefore, we followed the approach of Robben et al [23] to call SNPs across our cells in standard (ST) males. Briefly, using the cellranger generated BAM files for each sample, duplicate reads were marked and removed using GATK’s MarkDuplicates, reads with Ns in their cigar string were split with SplitNCigarReads and finally, SNPs were called using HaplotypeCaller. Variants were then filtered for a minGQ of 20, minDP of 4 and minQ of 30. Using this set of SNPs, we identified single-cell level variants using scAlleleCount (https://github.com/barkasn/scAlleleCount). We then calculated the level of heterozygosity for each cell as an estimate of ploidy, classifying cells as haploid if homozygous at > 95% of SNPs.

As we observed a decrease in transcriptional activity over spermatogenesis, we first checked for any confounding relationship between coverage depth and heterozygosity level that might bias our ability to robustly distinguish haploid and diploid cell types. We focused on diploid cell types (somatic tissue, cyst, GSC/spermatogonia and primary spermatocytes) for this test. We found a negative and significant relationship (*p* < 0.0001, ρ = -0.65) where cells with less coverage exhibited lower heterozygosity (Figures S14a & S14b). This is likely due to reduced power to confidently identify the minor allele when coverage is low. Therefore, to mitigate this bias we significantly increased our filtering for SNP calling to require at <= 2 reads for homozygotes to be called, and 4 for heterozygotes (2 reads mapping to both ref and alt) and a cell requiring variant information at >= 10 SNPs. However, even with this strict filtering, whilst the significant relationship between coverage and heterozygosity was weaker (*p* < 0.0001, ρ = -0.25), we still failed to identify differences in ploidy across cell types where expected (i.e., between somatic cells and spermatids) (Figures S14c & S14d). We hypothesise several reasons for our inability to detect ploidy differences between cell types, discussed below.

First, levels of heterozygosity in our population might be generally low because of inbreeding. This would lead to the incorrect assignment of diploid cells as haploid. We investigated this using DNA-sequencing data from nine standard individuals collected from the same population (Supplementary Methods). However, levels of heterozygosity are high and there is no evidence of inbreeding (Table S13).

Second, the stringent filtering we used to mitigate our reduced power to identify the minor allele when coverage is low means we had very few cells and sites remaining from which to assign ploidy. Specifically, after filtering, there were 2,359 cells remaining from the 4,469 genotyped cells with relaxed filtering. This will significantly reduce our power to robustly identify haploid and diploid cells.

Finally, across much of eukaryotic life, from *Drosophila* to humans, the post-meiotic germline becomes close to transcriptionally inert [24] after a huge peak in activity in the primary spermatocytes [13,25]. Transcripts that are transcribed in primary spermatocytes can remain dormant in the cytoplasm of the cell to be transcribed later in spermatogenesis [12,20]. Furthermore, in many species (e.g., rats [26] and *Drosophila* [27]) the spermatids within a bundle are joined by large cytoplasmic bridges that enable the movement of mRNA [28], proteins [29] and structures as large as organelles [26]. This means the cytoplasmic content does not just reflect the transcriptomic activity of one particular cell but also, to an extent, that of those neighbouring it [26-28]. Thus, if SNP calling for ploidy determination is reliant on single-cell RNA-seq approaches, reads sequenced from these cells were transcribed not just from themselves but also from earlier diploid cell states and from their neighbours. This could therefore give the appearance of diploidy even if the underlying genomic ploidy is haploid.

In summary, whilst assigning ploidy offers a valuable and alternate route to defining cell types in principle, we urge caution when interpreting these results particularly in non-model organisms where marker genes are either not reliable or absent. The use of joint sequencing [30] or single-nucleus (snRNA-seq) approaches would help to circumvent this challenge (see below).

***Single-cell vs single-nuclei sequencing approaches***

The relative merits of scRNA-seq and snRNA-seq have been discussed both broadly [31], and in the context of the study of spermatogenesis (Raz et al., 2023). Importantly, general patterns of expression during sperm development appear relatively consistent between methods [12]. An important consideration for using snRNA-seq in organisms with gene rich sex chromosomes may be the inability to detect X-linked cell type markers in droplets containing nuclei from haploid Y-carrying cells. This may be especially relevant in non-model organisms such as *Teleopsis dalmanni* where the pool of markers may be considerably smaller compared to species such as Drosophila.

Additionally, whilst snRNA-seq may capture transcriptional bursts that can be helpful in indicating later germ cells such as maturing spermatids, single-cell data provides a broader view of the available mRNA to the cell and those transcripts shared with it.

***Differential expression across unknown clusters***

There were five clusters which we were unable to classify and removed from subsequently analyses (Figure S13). Unknown clusters 3 and 5 were removed because they were only predominantly represented by a single sample. We therefore do not think these are biologically relevant. Unknown clusters 1, 2 and 4 were removed because they had no clear marker gene expression and so we were unable to assign a cell type.

To ensure we were not missing any important candidate genes, we conducted a differential expression analysis between standard and drive males for Unknown clusters 1, 2 and 4. For Unknown cluster 1, there were two differentially expressed genes (both SR-biased, *PB.523* and *grk*). For Unknown cluster 2, there were no differentially expressed genes. For Unknown cluster 4, there were two differentially expressed genes (one SR- and one ST-biased, *g459* and *Ced-12* respectively). With the exception of *Ced-12*, which we already identified as differentially expressed in early and late spermatids (Table S6), none of these genes are obvious candidates for the disruption of spermatogenesis.

**REFERENCES**

1. Li H, Durbin R. Fast and accurate short read alignment with Burrows–Wheeler transform. Bioinformatics. 2009;25(14): 1754–1760.
2. Danecek P, Auton A, Abecasis G, Albers CA, Banks E, DePristo MA, et al. The variant call format and VCFtools. Bioinformatics. 2011;27(15): 2156–2158.
3. van der Auwera G, O’Connor BD. Genomics in the Cloud: Using Docker, GATK, and WDL in Terra. O’Reilly Media, Incorporated. 2020.
4. Danecek P, Bonfield JK, Liddle J, Marshall J, Ohan V, Pollard MO, et al. Twelve years of SAMtools and BCFtools. GigaScience. 2021;10(2): giab008
5. Bonfield JK, Marshall J, Danecek P, Li H, Ohan V, Whitwham A, et al. HTSlib: C library for reading/writing high-throughput sequencing data. GigaScience. 2021;10(2): giab007
6. Emms DM, Kelly S. OrthoFinder: phylogenetic orthology inference for comparative genomics. Genome Biol. 2019;20(1): 238.
7. Löytynoja A, Goldman N. Phylogeny-aware gap placement prevents errors in sequence alignment and evolutionary analysis. Science. 2008;320(5883): 1632–1635.
8. Harrison PW, Jordan GE, Montgomery SH. SWAMP: Sliding Window Alignment Masker for PAML. Evol Bioinform Online. 2014;10: 197–204.
9. Yang Z. PAML 4: phylogenetic analysis by maximum likelihood. Mol Biol Evol. 2007;24(8): 1586–1591.
10. Li H, Janssens J, De Waegeneer M, Kolluru SS, Davie K, Gardeux V, et al. Fly Cell Atlas: A single-nucleus transcriptomic atlas of the adult fruit fly. Science. 2022;375(6584): eabk2432.
11. Mahadevaraju, S, Fear JM, Akeju M, Galletta BJ, Pinheiro MMLS, Avelino CC, et al. Dynamic sex chromosome expression in Drosophila male germ cells. Nat Commun. 2021;12(1): 892.
12. Raz AA, Vida GS, Stern SR, Mahadevaraju S, Fingerhut JM, Viveiros JM, et al. Emergent dynamics of adult stem cell lineages from single nucleus and single cell RNA-Seq of Drosophila testes. eLife. 2023;12: e82201.
13. Witt E, Benjamin S, Svetec N, Zhao L. Testis single-cell RNA-seq reveals the dynamics of de novo gene transcription and germline mutational bias in Drosophila. eLife. 2019;8: e47138
14. Ohlstein B, McKearin D. Ectopic expression of the Drosophila Bam protein eliminates oogenic germline stem cells. Development. 1997;124(18): 3651–3662.
15. Hess NK, Singer PA, Trinh K, Nikkhoy M, Bernstein SI. Transcriptional regulation of the *Drosophila melanogaster* muscle myosin heavy-chain gene. Gene Expr Patterns. 2007;7(4): 413–22.
16. Zoller R, Schulz C. The Drosophila cyst stem cell lineage: Partners behind the scenes? Spermatogenesis. 2012;2(3): 145–157.
17. Terry NA, Tulina N, Matunis E, DiNardo S. Novel regulators revealed by profiling Drosophila testis stem cells within their niche. Dev Biol. 2006;294(1): 246–257.
18. Vedelek V, Laurinyecz B, Kovács AL, Juhász G, Sinka R. Testis-Specific Bb8 Is Essential in the Development of Spermatid Mitochondria. PLoS One. 2016;11(8): e0161289.
19. Courtot C, Fankhauser C, Simanis V, Lehner CF. The Drosophila cdc25 homolog twine is required for meiosis. Development. 1992;116(2): 405–416.
20. Barreau C, Benson E, White-Cooper H. Comet and cup genes in Drosophila spermatogenesis: the first demonstration of post-meiotic transcription. Biochem Soc Trans. 2008;36(Pt 3): 540–542.
21. Baker CC, Gim BS, Fuller MT. Cell type-specific translational repression of Cyclin B during meiosis in males. Development. 2015;142(19): 3394–3402.
22. Santos IB, Wainman A, Garrido-Maraver J, Pires V, Riparbelli MG, Kovács L, et al. Mob4 is essential for spermatogenesis in *Drosophila melanogaster*. Genetics. 2023;224(4): iyad104
23. Robben M, Ramesh B, Pau S, Meletis D, Luber J Demuth J. scRNA-seq Reveals Novel Genetic Pathways and Sex Chromosome Regulation in Tribolium Spermatogenesis. Genome Biol Evol. 2024;16(3): evae059
24. Erickson RP. Post-meiotic gene expression. Trends Genet. 1990;6: 264–268.
25. Xia B, Yan Y, Baron M, Wagner F, Barkley D, Chiodin M, et al. Widespread Transcriptional Scanning in the Testis Modulates Gene Evolution Rates. Cell. 2020;180(2): 248–262.e21.
26. Ventelä S, Toppari J, Parvinen M. Intercellular Organelle Traffic through Cytoplasmic Bridges in Early Spermatids of the Rat: Mechanisms of Haploid Gene Product Sharing. Mol Biol Cell. 2003;14(7): 2768–2780.
27. Greenbaum MP, Iwamori T, Buchold GM, Matzuk MM. Germ Cell Intercellular Bridges. Cold Spring Harb Perspect Biol. 2011;3(8): a005850
28. Braun RE, Behringer RR, Peschon JJ, Brinster RL, Palmiter RD. Genetically haploid spermatids are phenotypically diploid. Nature. 1989;337(6205): 373–376.
29. Kaufman RS, Price KL, Mannix KM, Ayers KM, Hudson AM, Cooley L. Drosophila sperm development and intercellular cytoplasm sharing through ring canals do not require an intact fusome. Development. 2020;147(22): dev190140.
30. Vandereyken K, Sifrim A, Thienpont B, Voet T. Methods and applications for single-cell and spatial multi-omics. Nat Rev Genet. 2023;24(8): 494–515.
31. Ding J, Adiconis X, Simmons SK, Kowalczyk MS, Hession CC, Marjanovic ND, et al. Systematic comparison of single-cell and single-nucleus RNA-sequencing methods. Nat Biotechnol. 2020;38(6): 737–746.
