# Supplementary figures and images for: Single-cell consequences of X-linked meiotic drive in stalk-eyed flies
Source: PLoS Genet. 2025 Sep 18;21(9):e1011816. doi: 10.1371/journal.pgen.1011816 (PMC12445520; doi:10.1371/journal.pgen.1011816)

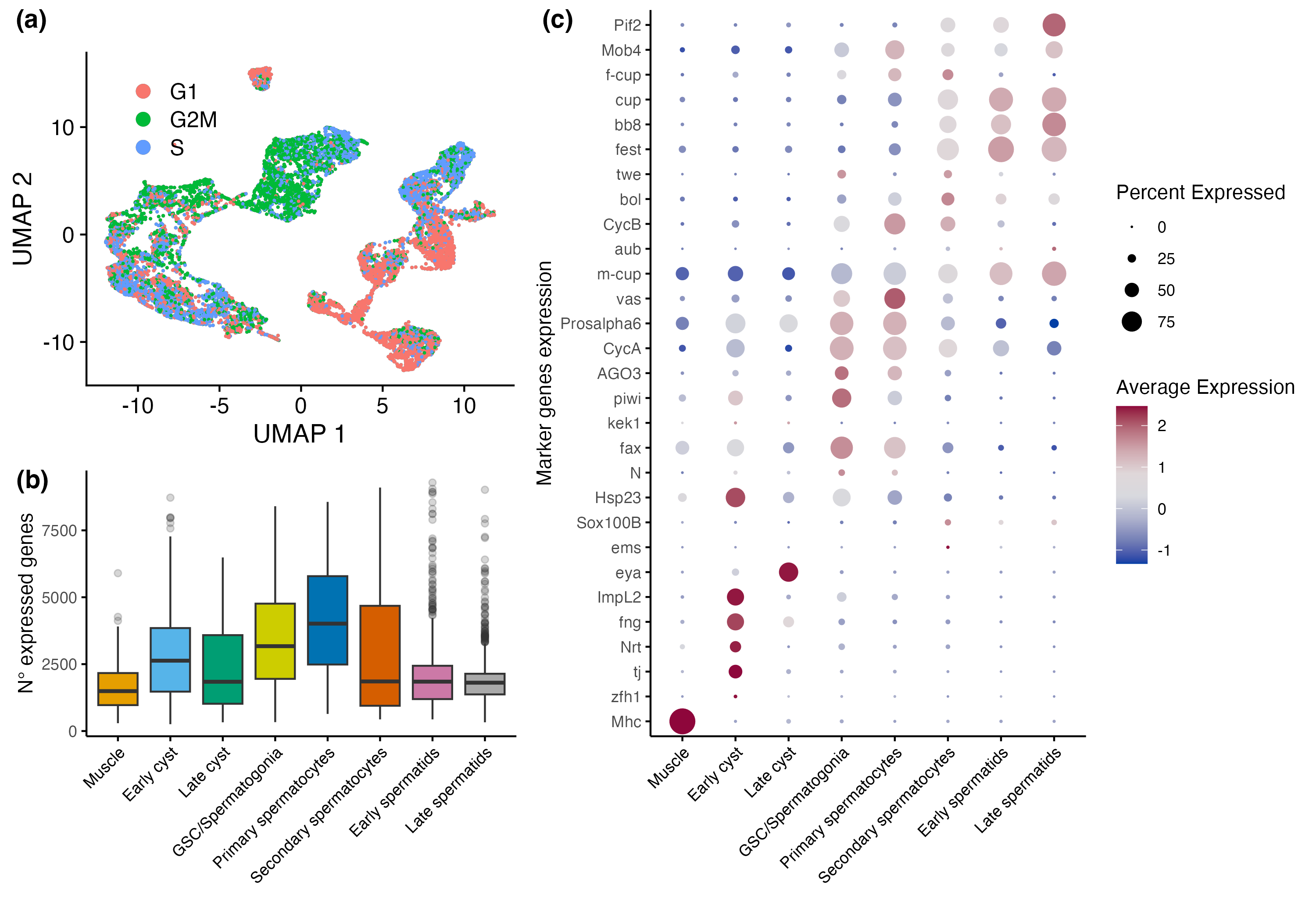

Supplement: S1 Fig — (A) Uniform Manifold Approximation and Projection (UMAP) of cells classified by mitotic stage marker expression as a proxy for cell cycle stage. G1: Gap 1, S: DNA synthesis, G2M: Gap 2/Mitosis (B) Boxplots of number of autosomal genes expressed across all cell types (gene classified as expressed if counts > 1). (C) Dot plot of relative expression of orthologs of Drosophila melanogaster cell-type-specific testis markers. Size of dots indicates the relative number of cells expressing the marker in a cluster and colour indicates the level of expression (blue lowest and red highest). (TIFF) [file pgen.1011816.s002.tiff]

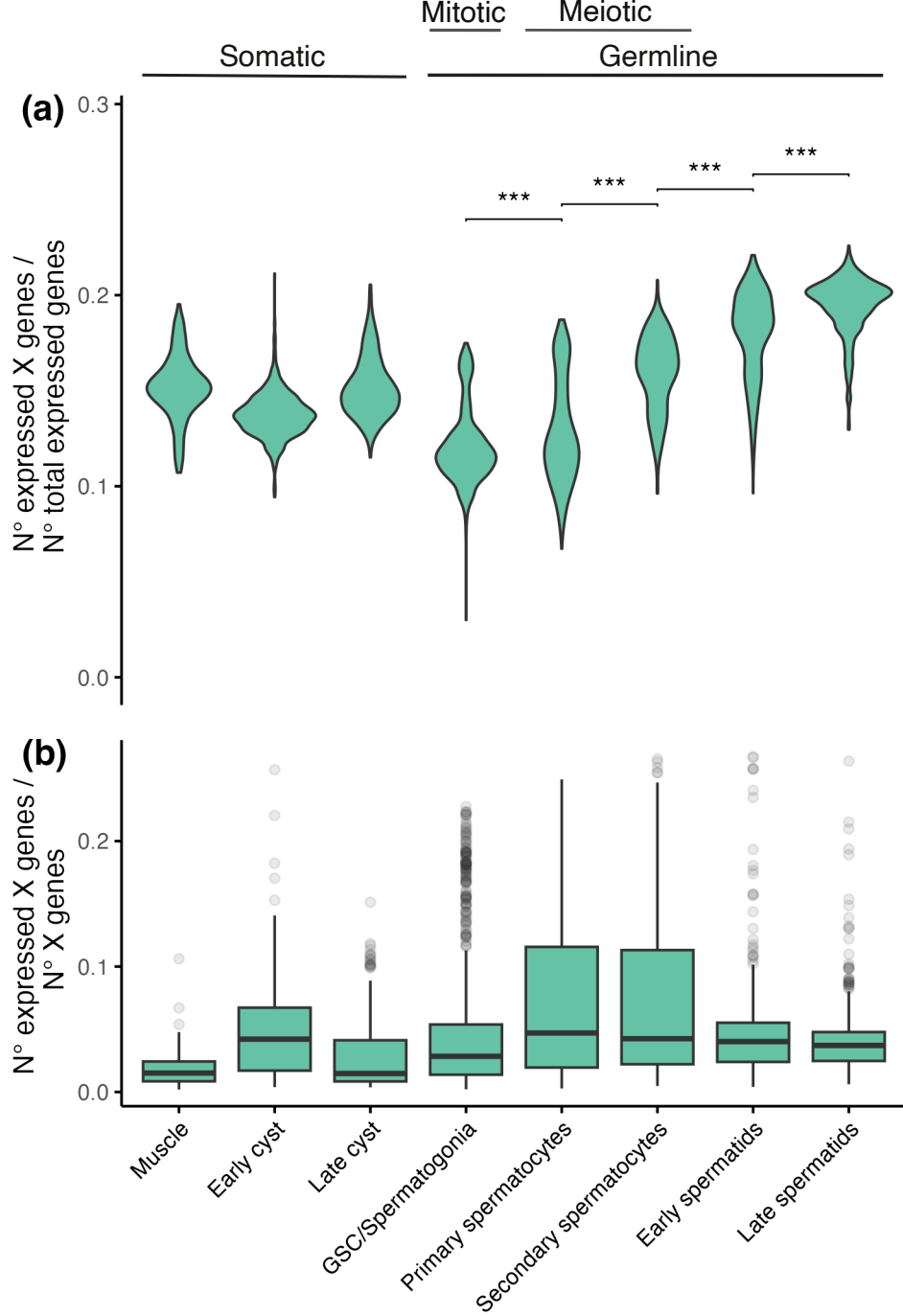

Supplement: S2 Fig — Violin plot showing the relative number of X-linked genes expressed across T. dalmanni cell types in standard (ST) males. A two-sided Wilcoxon test was used to determine if values in (A) differed across the stages of spermatogenesis where p < 0.00001 = ***, p < 0.001 = **, p < 0.05 = *. (PDF) [file pgen.1011816.s003.pdf]

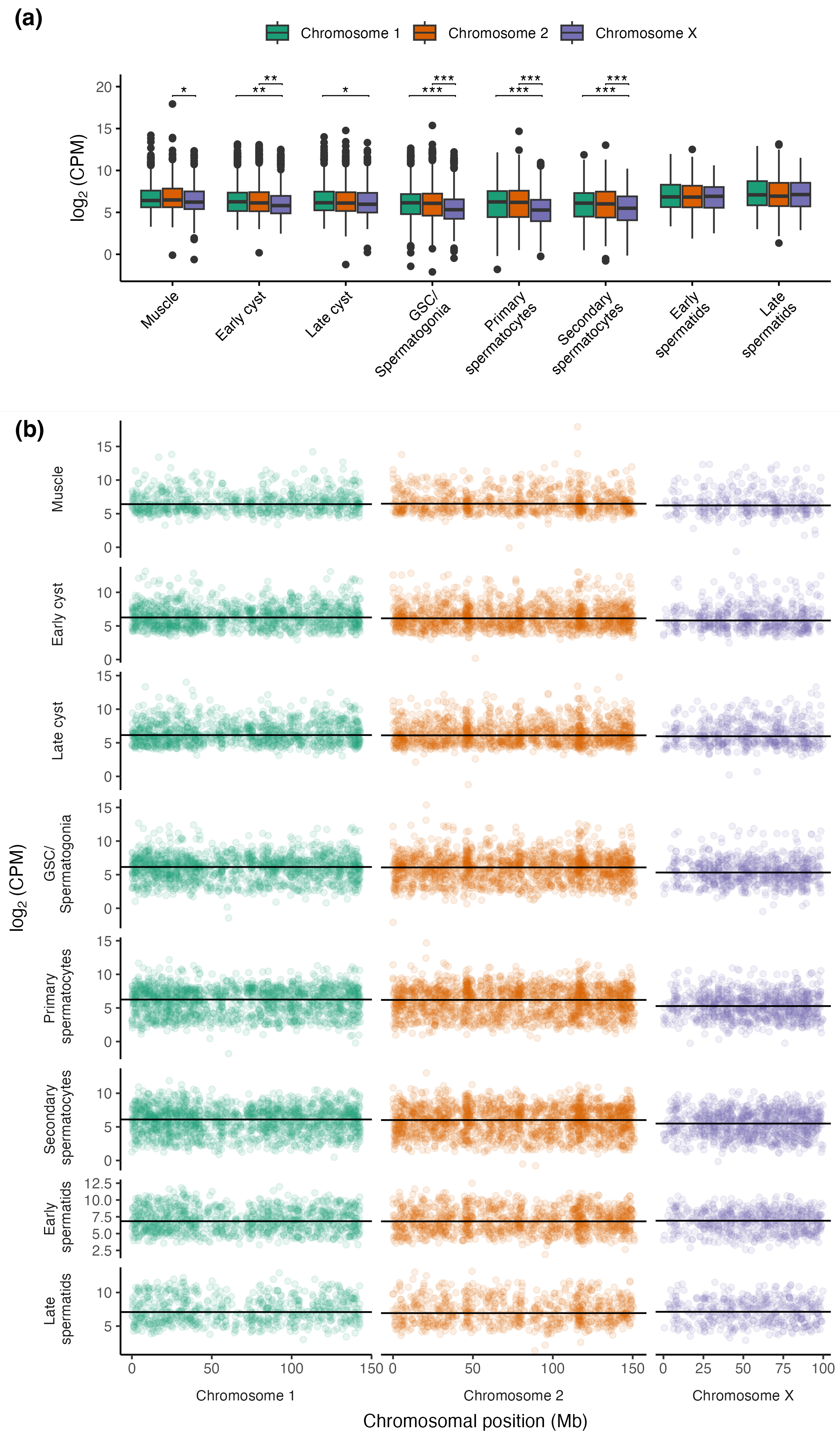

Supplement: S3 Fig — (A) Expression values of genes in standard (ST) males from chromosome 1, 2 and the X chromosome across cell types. Expression values were compared in a pairwise manner between chromosomes with a two-sided Wilcoxon test. p < 0.00001 = ***, p < 0.001 = **, p < 0.05 = *. (B) Distribution of gene expression across the genome in each cell type. Expression is measured as the average log2(CPM) across ST males for each cell type, utilising a pseudobulk approach. (TIFF) [file pgen.1011816.s004.tiff]

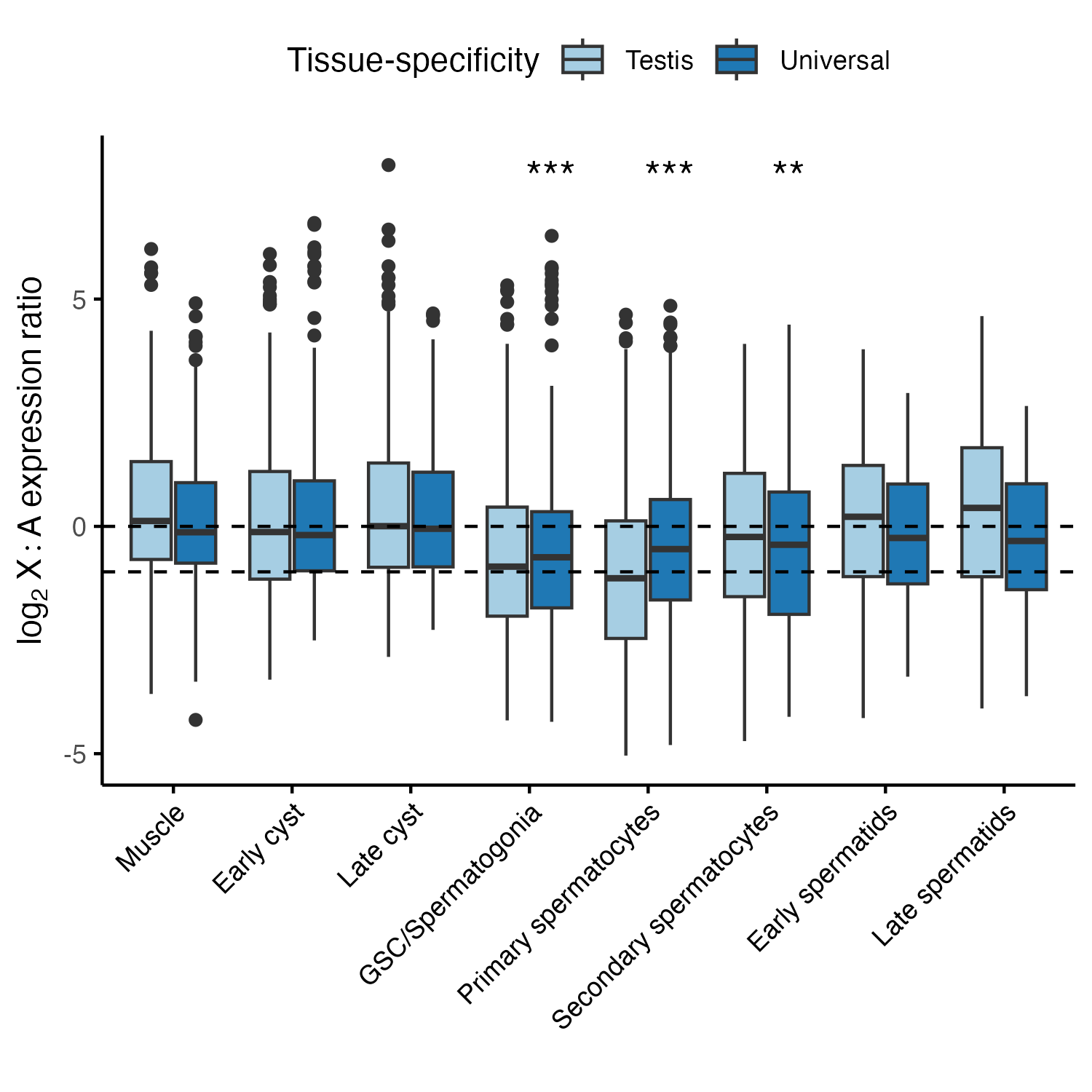

Supplement: S4 Fig — Box plots of the log2 ratio of X-linked gene expression to median autosomal expression, measured in counts per million (CPM), across cell types. A two-sided Wilcoxon test was used to determine if values for each cell type and class of tissue-specificity differed from 0. p < 0.00001 = ***, p < 0.001 = **, p < 0.05 = *. (TIFF) [file pgen.1011816.s005.tiff]

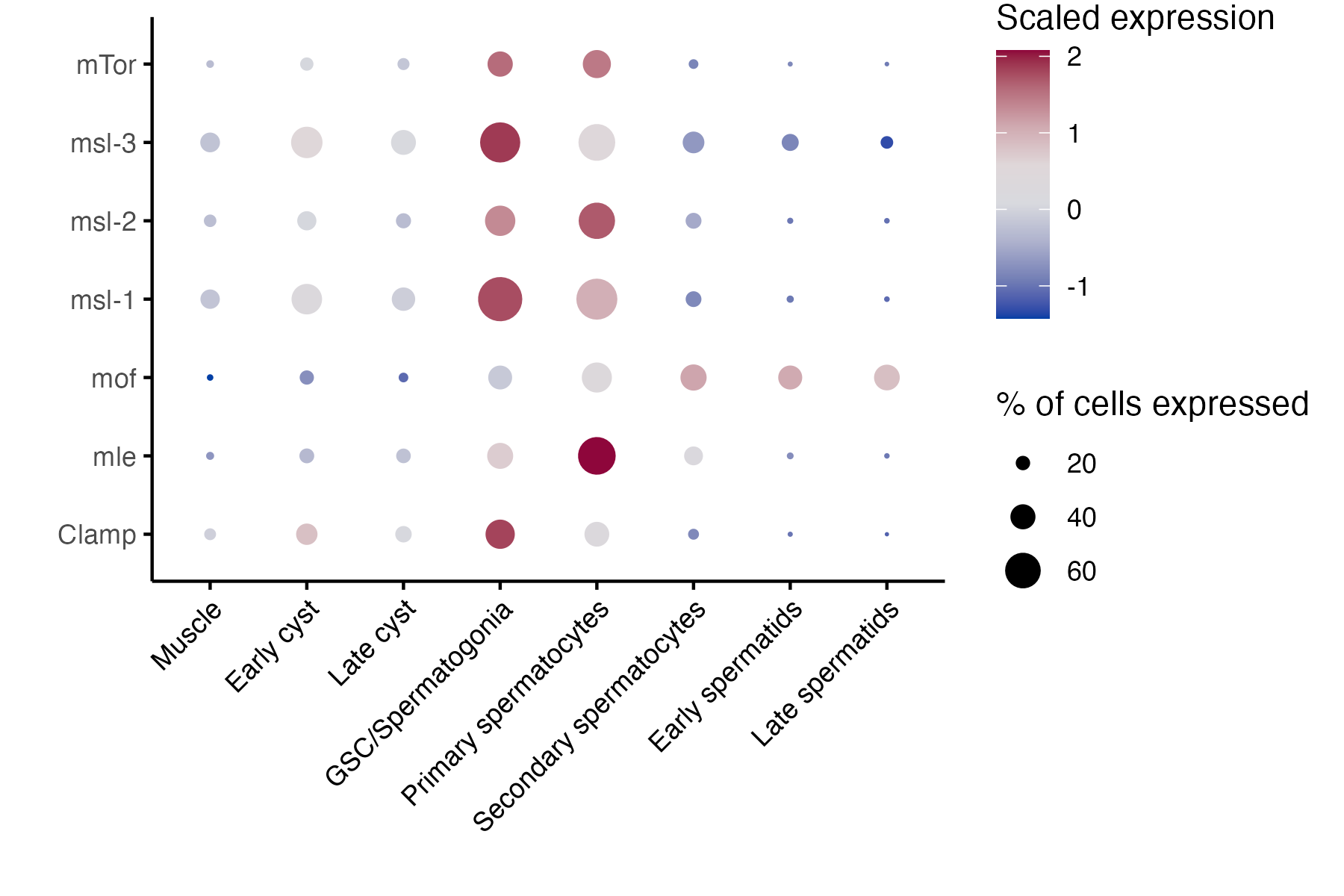

Supplement: S5 Fig — Dot plot of scaled expression of T. dalmanni orthologs of components and accessory genes of the dosage compensation complex (DCC) in Drosophila. Colour signifies scaled expression and dot size represents the percentage of cells expressing the specific gene. (TIFF) [file pgen.1011816.s006.tiff]

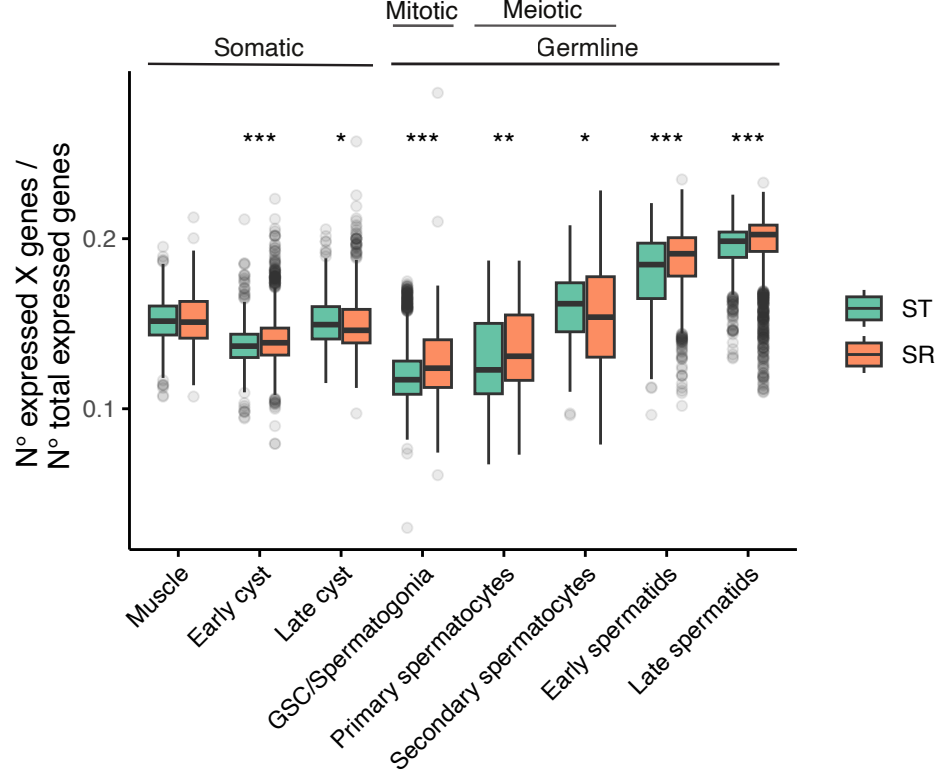

Supplement: S6 Fig — Box plot showing the relative number of X-linked genes expressed across cell types in standard (ST) and drive (SR) males. A two-sided Wilcoxon test was used to determine if the proportion of expressed X-linked genes for each cell type differed between ST and SR cells. p < 0.00001 = ***, p < 0.001 = **, p < 0.05 = *. (PDF) [file pgen.1011816.s007.pdf]

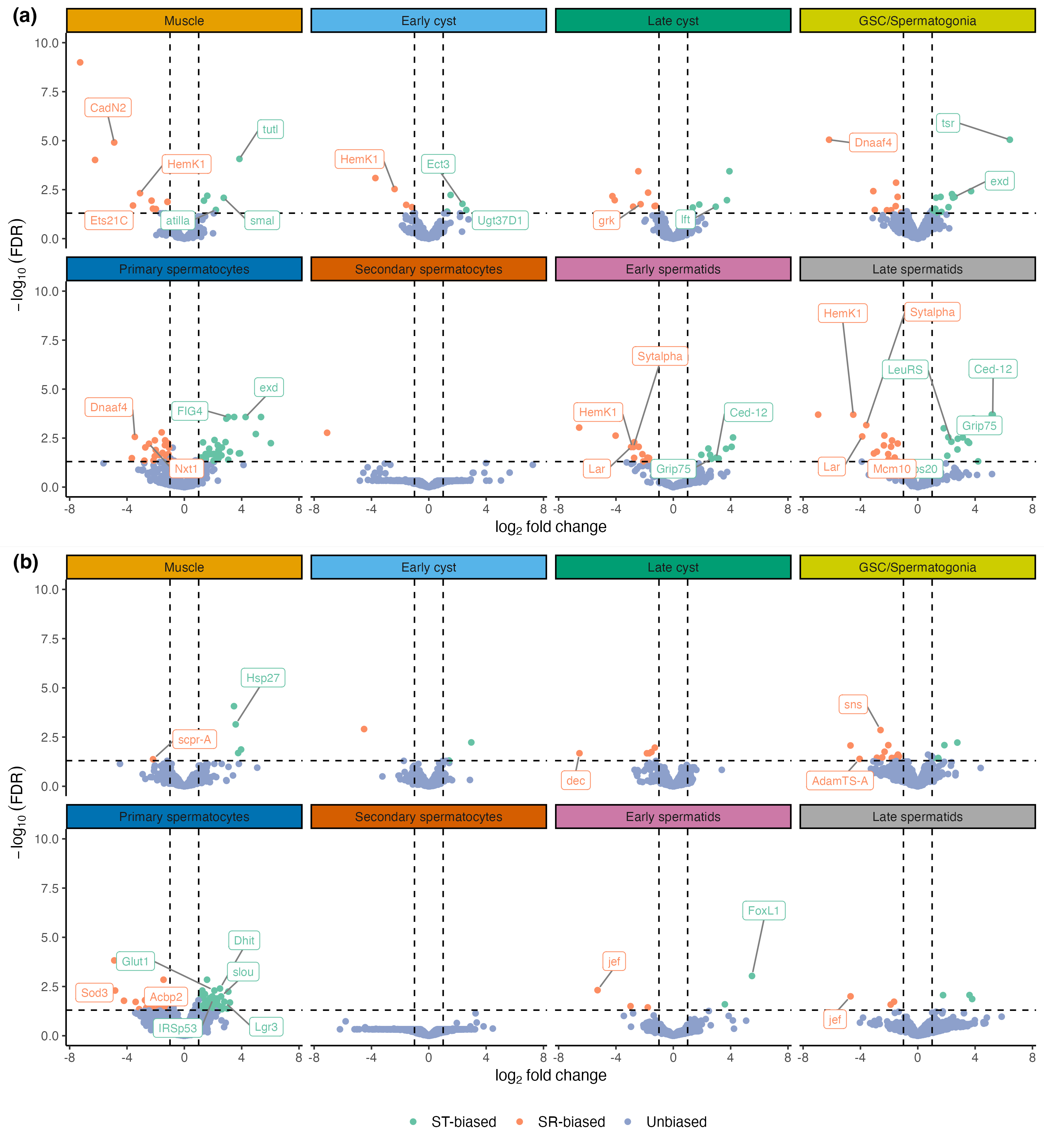

Supplement: S7 Fig — Volcano plots of differentially expressed genes in each cell type for (a) the X chromosome and (B) the autosomes with log2 fold-change on X axis and False Discovery Rate (FDR) adjusted p-value on the Y axis. Blue points are unbiased genes and green and orange dots are drive (SR-) and standard (ST-) biased genes respectively (FDR < 0.05 and absolute log2(fold-change) > 1). Labelled points are the top 8 significant genes per cell type (ordered by FDR) with Drosophila orthologs (Note that not all cell types have 8 genes matching this criteria). (TIFF) [file pgen.1011816.s008.tiff]

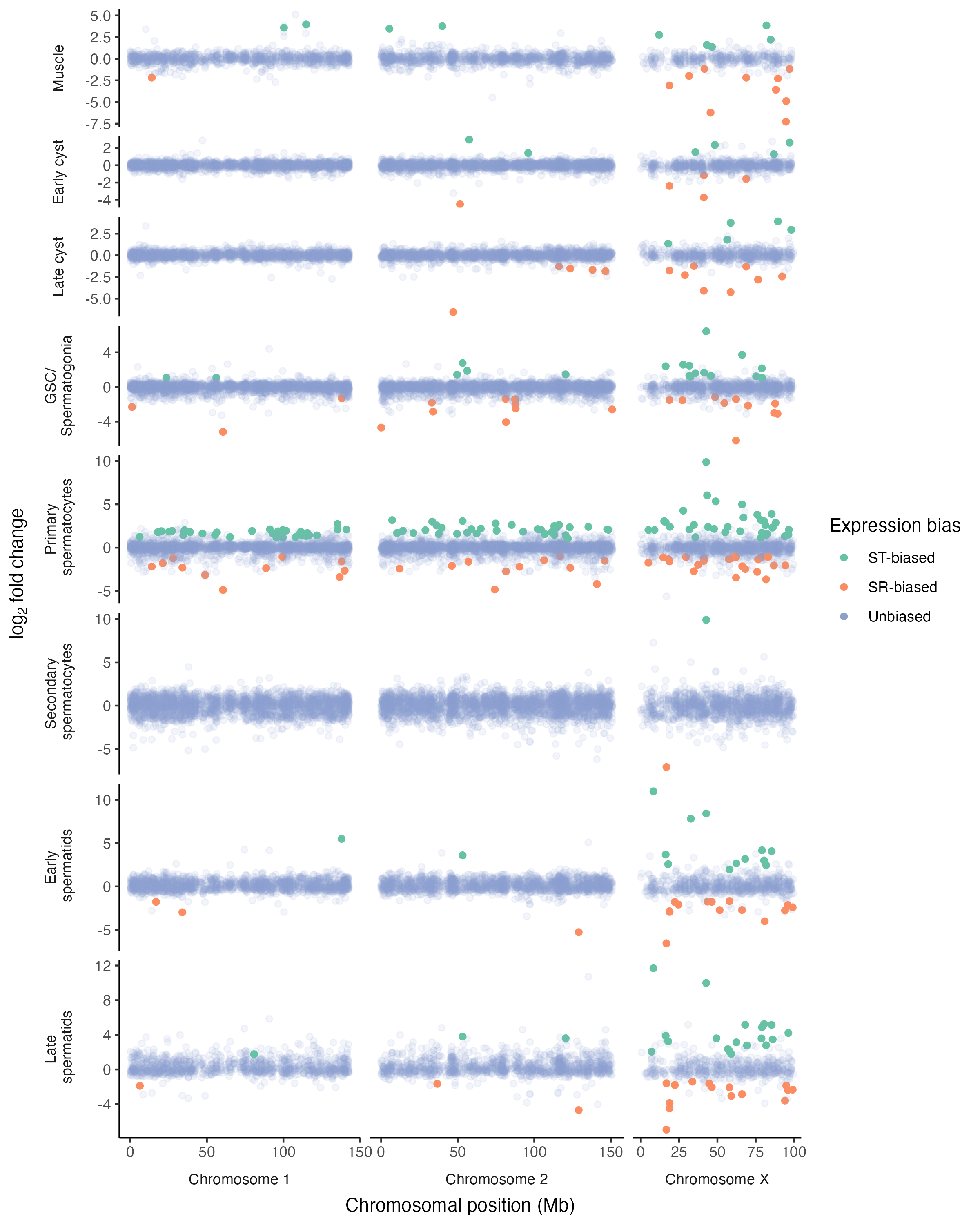

Supplement: S8 Fig — Dot plots of differentially expressed genes in each cell type across the genome with chromosomal position on X axis and log2 fold-change on Y axis. Blue points are unbiased genes and green and orange dots are drive (SR-) and standard (ST-) biased genes respectively (FDR < 0.05 and absolute log2(fold-change) > 1). (TIFF) [file pgen.1011816.s009.tiff]

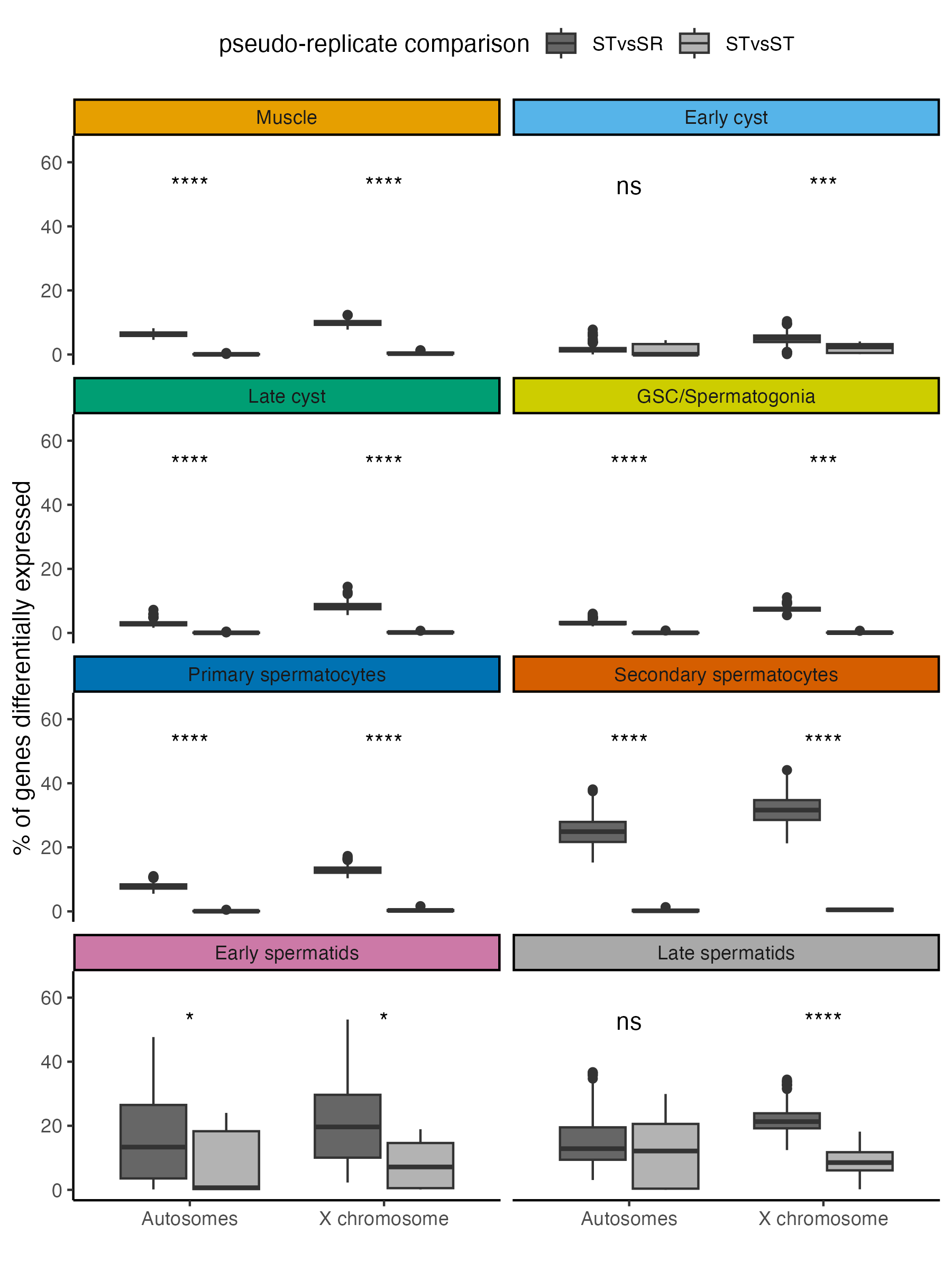

Supplement: S9 Fig — To validate enrichment of differential expression to the X, pseudo-samples were generated from the pool of ST and SR cells. For ST vs ST comparisons, two sets of four pseudo-samples of 100 cells were produced. These cells were sampled with replacement from the pool of all ST cells. The percentage of genes differentially expressed between each ST group was then calculated for the X and autosomes. The same approach was then applied to compare pseudo-samples between ST and SR, creating four pseudo-samples for ST and four for SR. A distribution of the % of differentially expressed genes in each comparison was generated through 1000 repeats. A Wilcoxon test was used to determine whether the proportion of genes differentially expressed in ST vs SR differed to ST vs ST. (TIFF) [file pgen.1011816.s010.tiff]

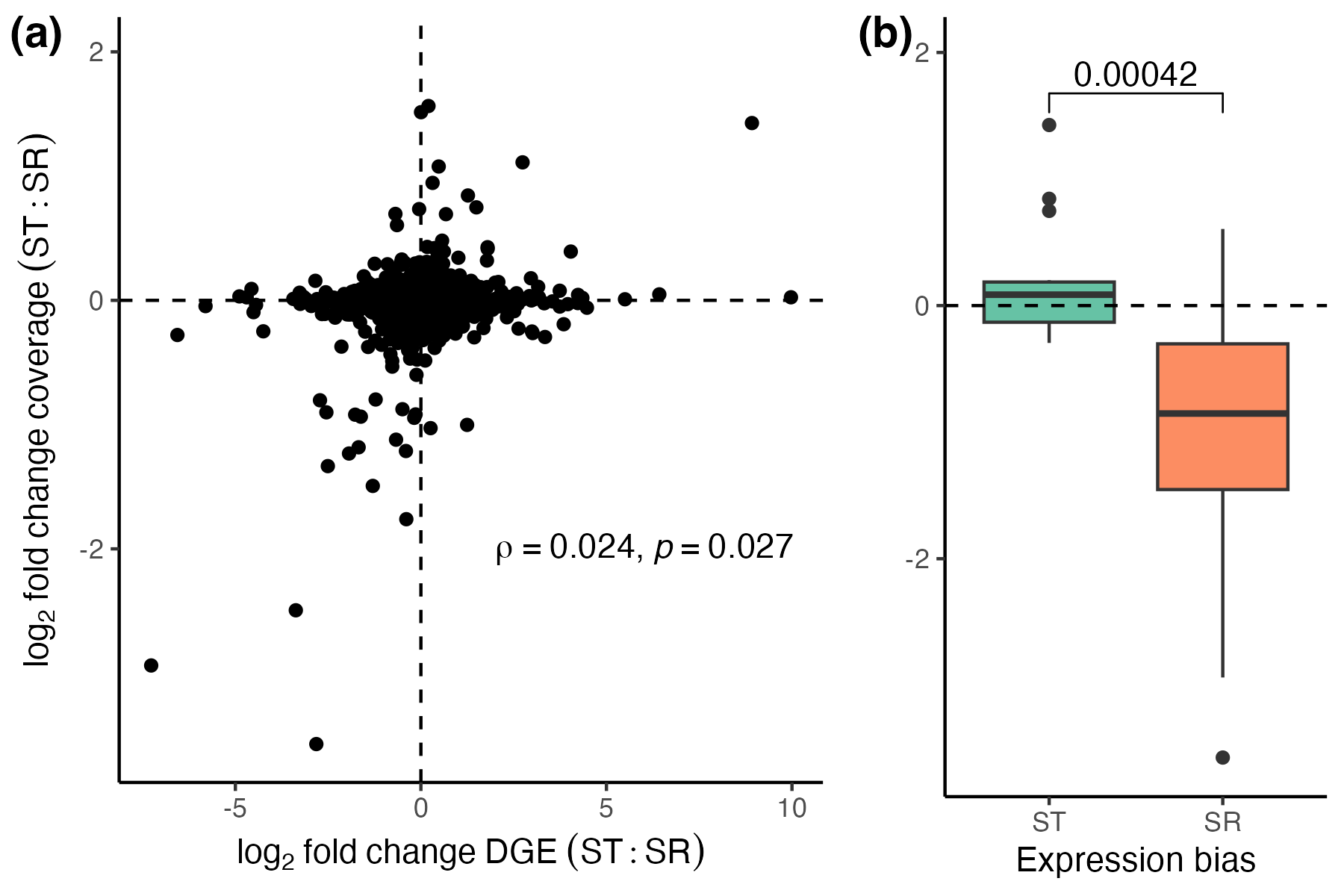

Supplement: S10 Fig — (A) Scatter plot of average log2 fold-change of expression against log2 fold-change in coverage between ST and SR. Relationship determined by a Spearman’s rank. (b) Boxplot comparing genes that exhibit significant (p < 0.05) differential expression and differential coverage (n = 28). For this set of genes, a Wilcoxon test was used to determine whether coverage varied between ST- and SR-biased genes. (TIFF) [file pgen.1011816.s011.tiff]

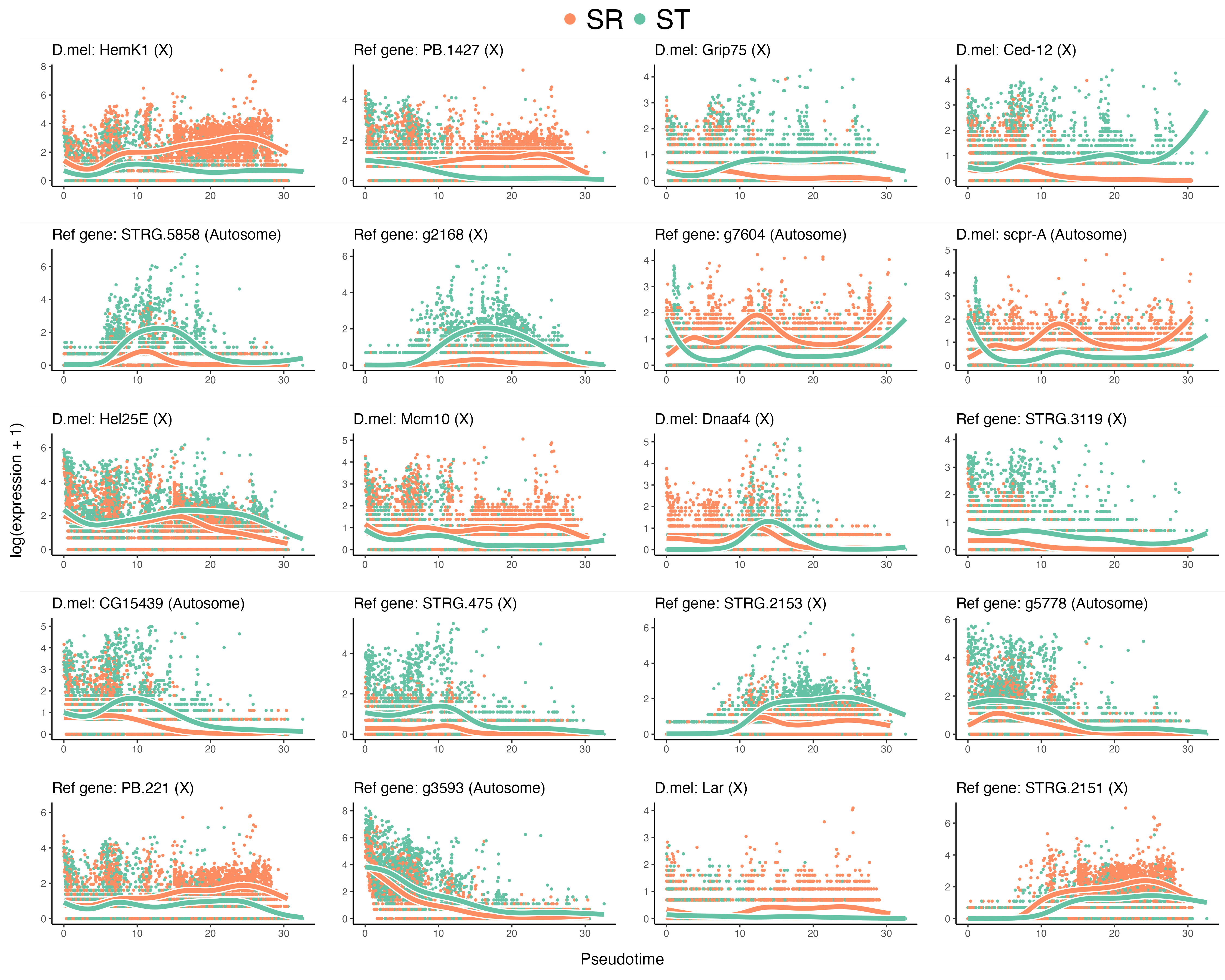

Supplement: S11 Fig — Differential trajectories for cells assigned to germline cell types (GSC/spermatogonia, primary and secondary spermatocytes, and spermatids). Plotted are genes identified as those that are both differentially expressed and with differential trajectories between standard (ST) and drive (SR) individuals (p-value < 0.05 & log2 fold-change > 2). Genes are then ordered by descending Wald stat from the condition test for identifying differential trajectories with the top 20 shown. D.mel refers to genes with Drosophila melanogaster orthologs, and Ref gene are those without. (TIFF) [file pgen.1011816.s012.tiff]

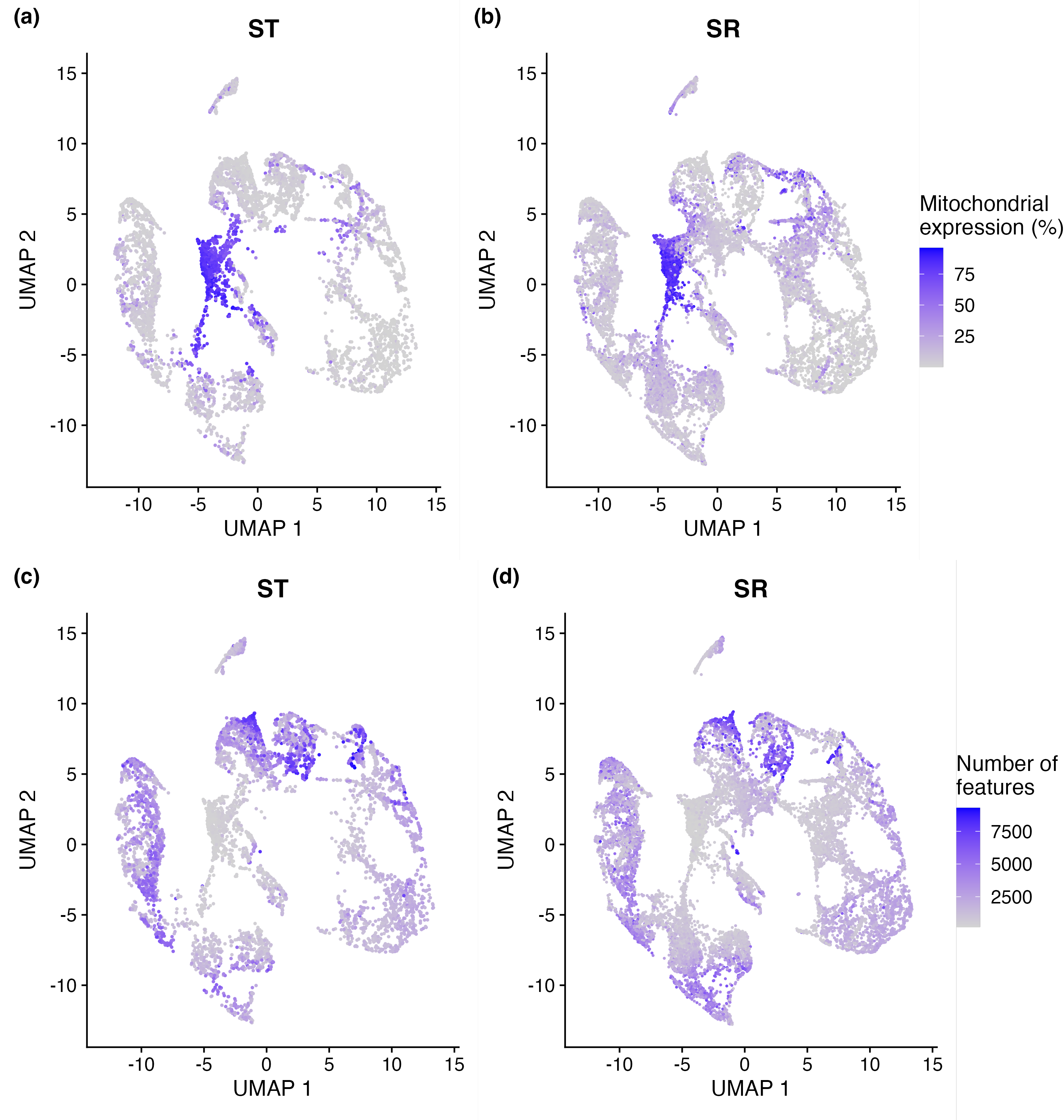

Supplement: S12 Fig — UMAP of raw data before filtering for mitochondrial expression or selection of cell types (a and b) and number of expressed features (c and d) for ST and SR samples. Colour intensity in (A) and (B) represents the level of mitochondrial expression measured as the percentage of transcripts in each cell mapping to the mitochondrial genome. Colour intensity in (c) and (d) represents the number of expressed features in each cell. 21% of SR cells and 19% of ST cells failed the 20% mitochondrial expression filter and were removed. Most cells that fail the 20% filter fall into a single cluster which is represented by both standard and drive cells. Notably, cells in this cluster also express very few genes (C and D), which is highly symptomatic of dying cells. Importantly, this cluster is a similar size between ST and SR. Therefore, we do not think it is a biologically real cluster of cells dying due to driver action but is instead an artifact where all dying cells across the testes have clustered together because of their unique expression profile (e.g., high mitochondrial expression and low no. of features). (TIFF) [file pgen.1011816.s013.tiff]

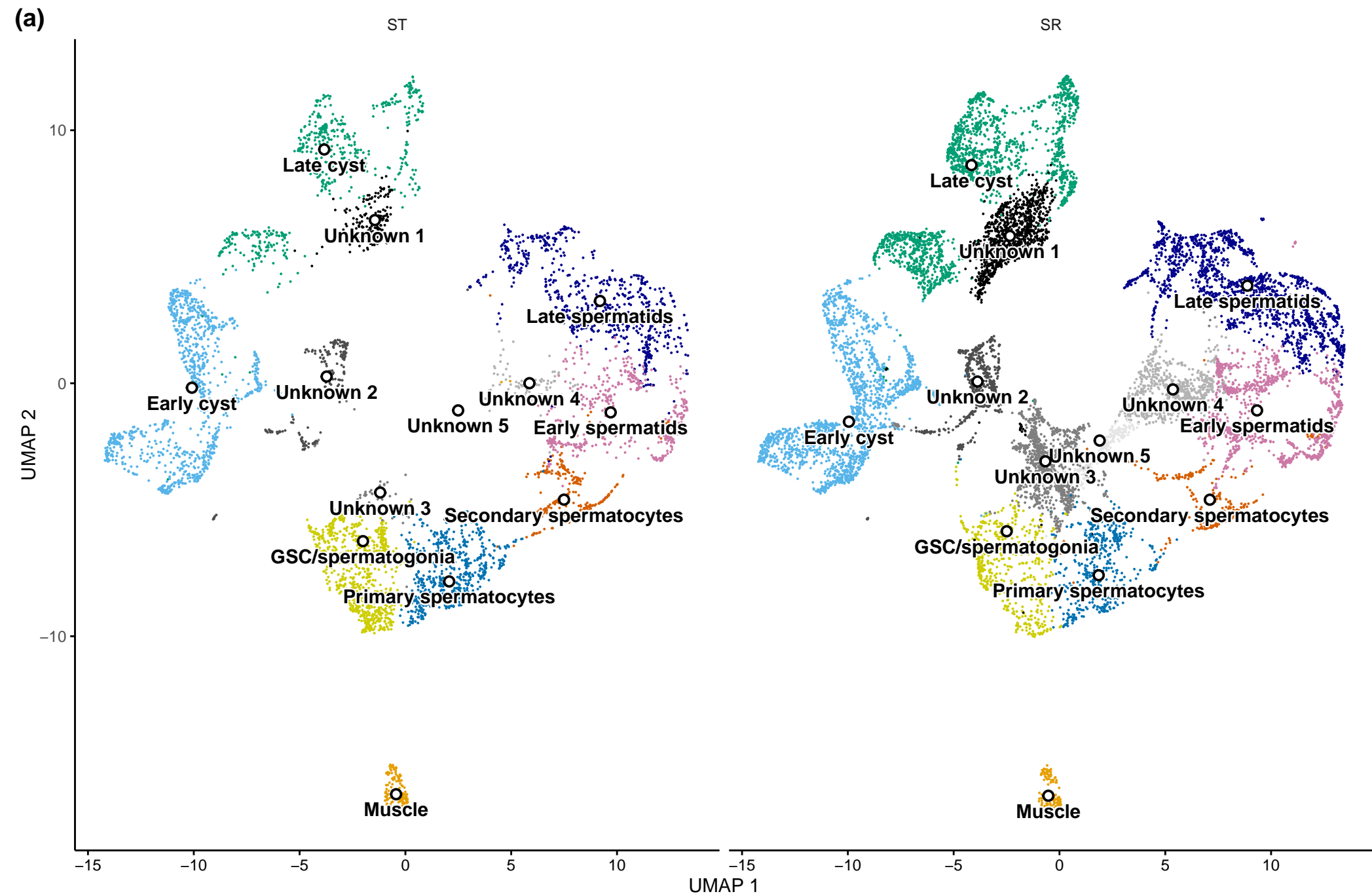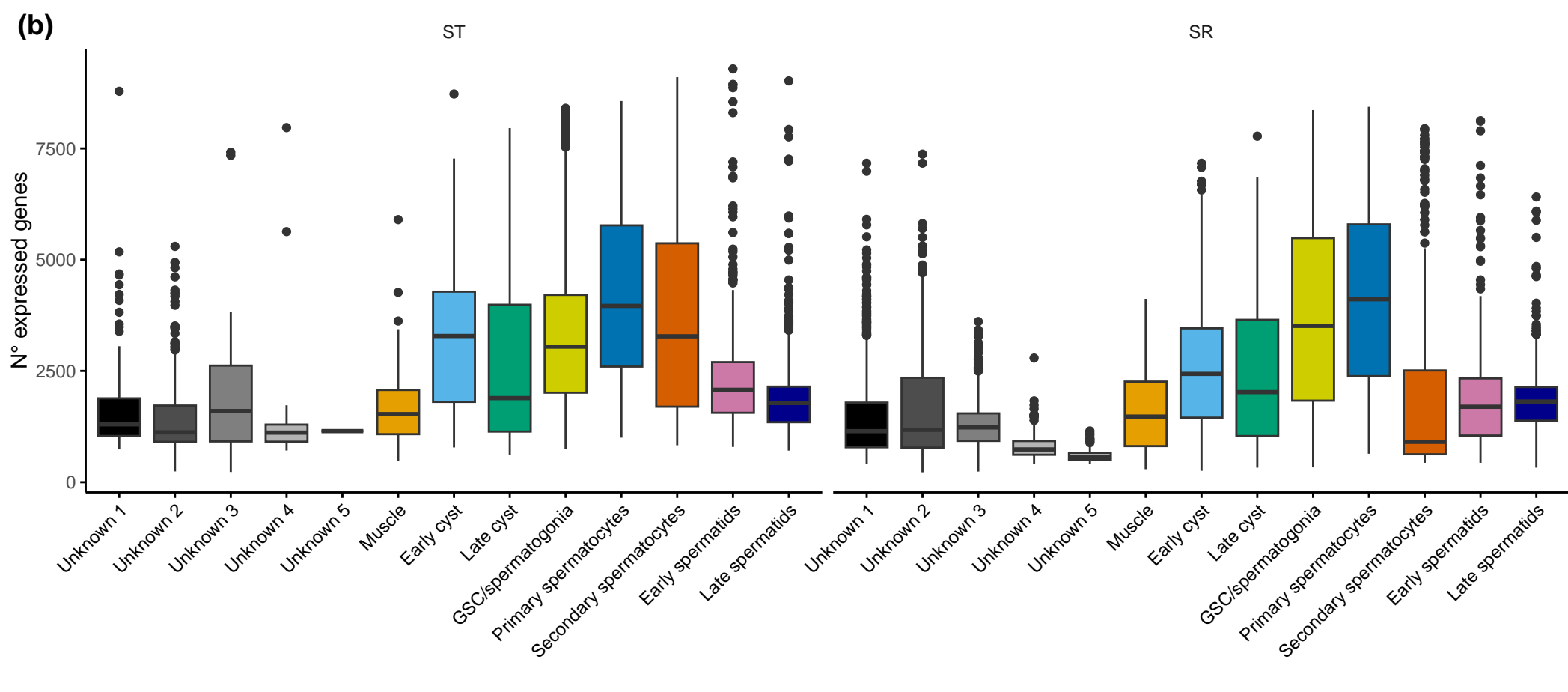

Supplement: S13 Fig — Single-cell data set before removal of clusters with no clear biological classification or that were predominantly represented by a single sample. (A) UMAP of identified cell types from unfiltered single-cell data sets for ST and SR samples. (B) Boxplots of number of genes expressed across cell types (gene classified as expressed if counts > 1). (PDF) [file pgen.1011816.s014.pdf]

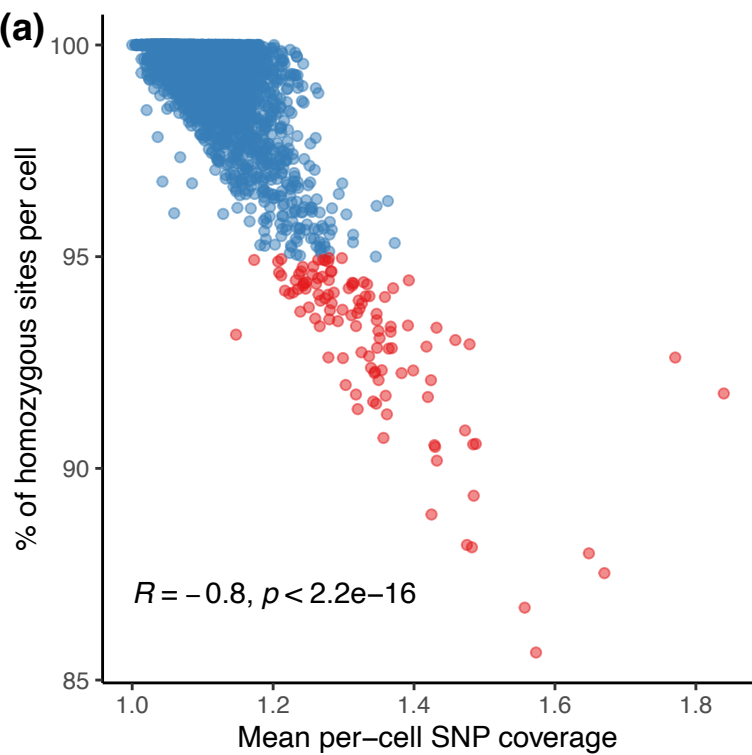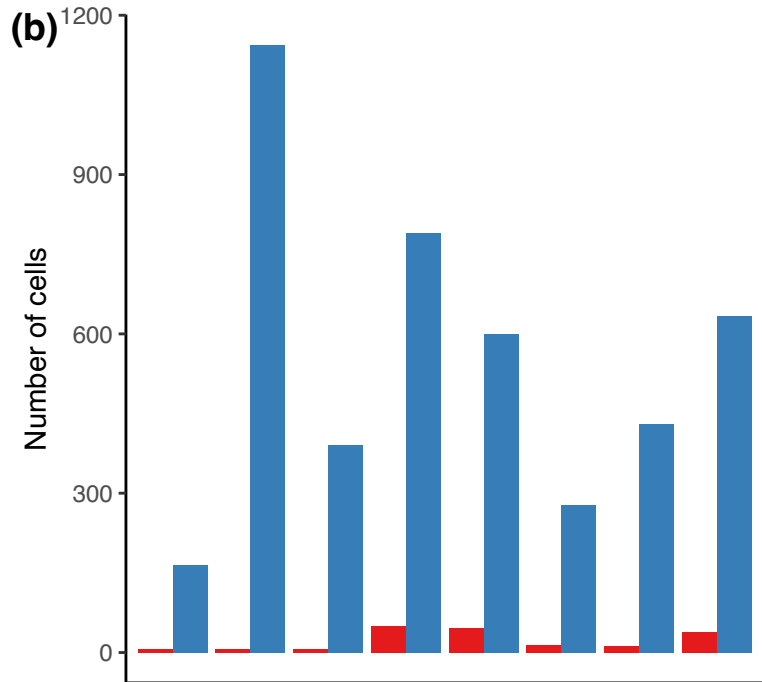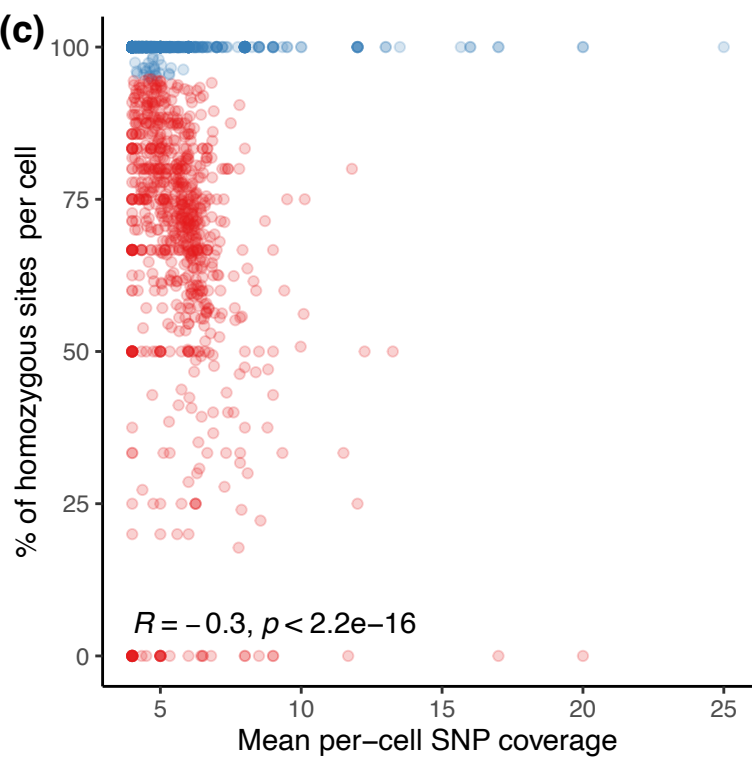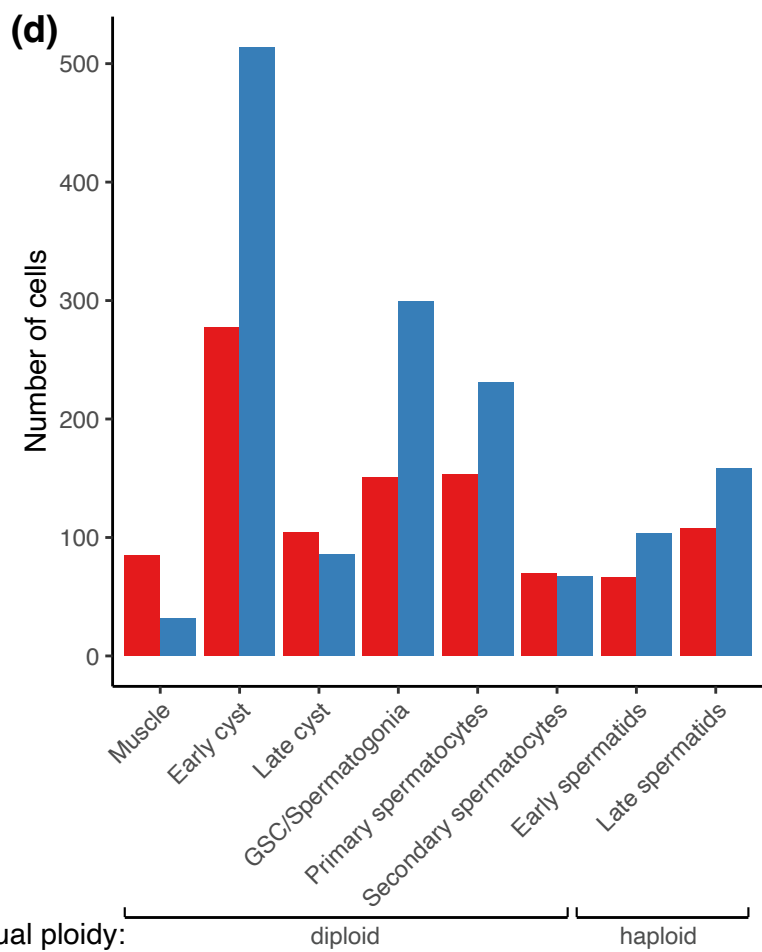

Supplement: S14 Fig — (A) Percentage of sites per cell that are homozygous (all reads matching either reference or alternate at a site). Data only shown for standard (ST) male diploid cell types (cyst, muscle, GSC/spermatogonia, and primary spermatocytes) No depth or minimum number of site thresholds were set for calling the ploidy of each cell. (B) Number of cells classified as haploid or diploid for each cell type following the filtering in (A). (C) and (D) are the same as (A) and (B) however a threshold of being genotyped at>= 10 sites per cell with depth of>= two for calling homozygous or four for heterozygous (two mapping to both ref and alt). (PDF) [file pgen.1011816.s015.pdf]
